# Evolutionary conservation and divergence of the human brain transcriptome

**DOI:** 10.1186/s13059-020-02257-z

**Published:** 2021-01-29

**Authors:** William G. Pembroke, Christopher L. Hartl, Daniel H. Geschwind

**Affiliations:** 1 Program in Neurogenetics, Department of Neurology, David Geffen School of Medicine, UCLA, Los Angeles, CA USA; 2 Center for Autism Research and Treatment, Semel Institute, David Geffen School of Medicine, UCLA, Los Angeles, CA USA; 3 Department of Human Genetics, David Geffen School of Medicine, UCLA, Los Angeles, CA USA

**Keywords:** Evolution, Genomics, Transcriptome, Co-expression, Neuroscience, Disease

## Abstract

**Background:**

Mouse models have allowed for the direct interrogation of genetic effects on molecular, physiological, and behavioral brain phenotypes. However, it is unknown to what extent neurological or psychiatric traits may be human- or primate-specific and therefore which components can be faithfully recapitulated in mouse models.

**Results:**

We compare conservation of co-expression in 116 independent data sets derived from human, mouse, and non-human primate representing more than 15,000 total samples. We observe greater changes occurring on the human lineage than mouse, and substantial regional variation that highlights cerebral cortex as the most diverged region. Glia, notably microglia, astrocytes, and oligodendrocytes are the most divergent cell type, three times more on average than neurons. We show that cis-regulatory sequence divergence explains a significant fraction of co-expression divergence. Moreover, protein coding sequence constraint parallels co-expression conservation, such that genes with loss of function intolerance are enriched in neuronal, rather than glial modules. We identify dozens of human neuropsychiatric and neurodegenerative disease risk genes, such as COMT, PSEN-1, LRRK2, SHANK3, and SNCA, with highly divergent co-expression between mouse and human and show that 3D human brain organoids recapitulate in vivo co-expression modules representing several human cell types.

**Conclusions:**

We identify robust co-expression modules reflecting whole-brain and regional patterns of gene expression. Compared with those that represent basic metabolic processes, cell-type-specific modules, most prominently glial modules, are the most divergent between species. These data and analyses serve as a foundational resource to guide human disease modeling and its interpretation.

**Supplementary Information:**

The online version contains supplementary material available at 10.1186/s13059-020-02257-z.

## Introduction

The human brain is the culmination of millions of years of evolution [1], showcasing high-level cognitive processes such as symbolic thought, self-reflection, and the ability for long-term planning. Much of our understanding of human brain and disease is derived from studies in mice [2]. However, extrapolation from mouse models may be limited because humans last shared a common ancestor with mice 90 million years ago (mya) [3, 4]. Humans exhibit a five-fold relative expansion in cortical volume, changes in cellular composition, and cell-type differences, such as a substantial increase in size and complexity of human astrocytes [5–7]. Furthermore, many distinctions, such as the molecular reorganization of specific cell types and neural circuits have arisen over recent human evolution [1]. A number of neurological disorders are associated with the dysfunction of biological processes that occur in brain regions or cell types that possess features specific to human biology [8–10], posing the question as to which components underlying human neuropsychiatric diseases can be faithfully recapitulated in model organisms, or for that matter, cell-based model systems [11].

Human evolution is hypothesized to be driven primarily by changes in gene regulation rather than protein sequence divergence [12–14], highlighting the transcriptome as an appropriate nexus for investigating evolution, as demonstrated by recent studies assessing gene expression in brains across multiple primate species [15, 16]. While many gene expression changes may be a result of neutral drift over the course of evolution [17], gene co-expression networks provide a functional framework for assessing whether changes in expression are indeed neutral, or have a functional impact [18]. Gene co-expression networks built on data from human brain tissues have been utilized to assess which aspects of human brain function are preserved and diverged across species [8, 12, 18]. These studies were either conducted using small sample sizes from a single, or a few brain regions, or constructed “brain-wide” co-expression networks, which limits the identification of more subtle “intra-region” transcriptomic patterns.

Due to natural variation in cell-type proportion across tissue samples, co-expression analysis using homogenous tissue with large sample sizes allows us to generate cell-type information without the need to physically isolate cells, so called “in silico dissection” [19, 20]. Single-cell sequencing of both human and mouse cortex has allowed identification of species differences at a cell-type resolution [7]. However, this analysis was limited to a single brain region and focuses on differential gene expression within each cell class. Because co-expression reflects functional mechanisms such as co-regulation, changes in network position reflect changes in function [18, 21]. Therefore, investigating co-expression networks from multiple brain regions across different phylogenetic groups may allow us to assess the functional divergence of biological processes and cell types for many brain regions.

To identify robust evolutionarily divergent brain regions and biological processes, we constructed co-expression networks utilizing a bootstrapping approach for 12 brain regions in human based on the GTEx resource [22, 23] and 7 brain regions in mouse from 30 studies (Additional files 2,3: Table S1, S2). We assessed network divergence for each brain region in human and mouse by performing module preservation in the corresponding brain region for human, non-human primate (NHP), and mouse. Our analysis was in line with previous findings, with glial co-expression modules on average three times as divergent as neuronal modules [8, 19]. By exploring many major regions across the brain, we were able to identify regional variation and compare preservation across regions. We observed that cerebral cortical brain regions display the greatest relative divergence in human, an analysis enabled by the extensive regional sampling within the GTEx dataset [22]. We identify 5473 genes that display significant divergence of co-expression from human to mouse in at least one brain region, including many that have been related to human neurological and psychiatric disease.

By examining the relationship of co-expression divergence with measures of divergence at the DNA sequence level, including regulatory sequence changes and protein coding sequence constraint (pLI; [24]), we identify genetic mechanisms underlying divergence and show that the extent of transcriptomic divergence reflects other known measures of selection. Since co-expression modules are often related to cell type [19, 20], these divergent genes can begin to explain the nature of cell-type divergence between human and mouse. We show that gene divergence in co-expression is associated with changes in mean expression between species. Through integration with single-cell sequencing data, we show that these species differences in gene expression are largely due to cellular differences in gene expression rather than cell-type proportion differences in bulk tissue. Of these diverged genes, 91 (2%) show evidence of genetic association to at least one brain disorder, such as the autism (ASD) risk genes SCN2A and SHANK3. Furthermore, a substantial proportion (18%) of genes that display up- or downregulation in post-mortem brain of patients with neurological disorders display significant divergence of co-expression from human to mouse, potentially limiting their use as disease biomarkers in mouse.

## Results

### Assessing the evolutionary divergence of human and mouse brain networks

**Fig. 1:**
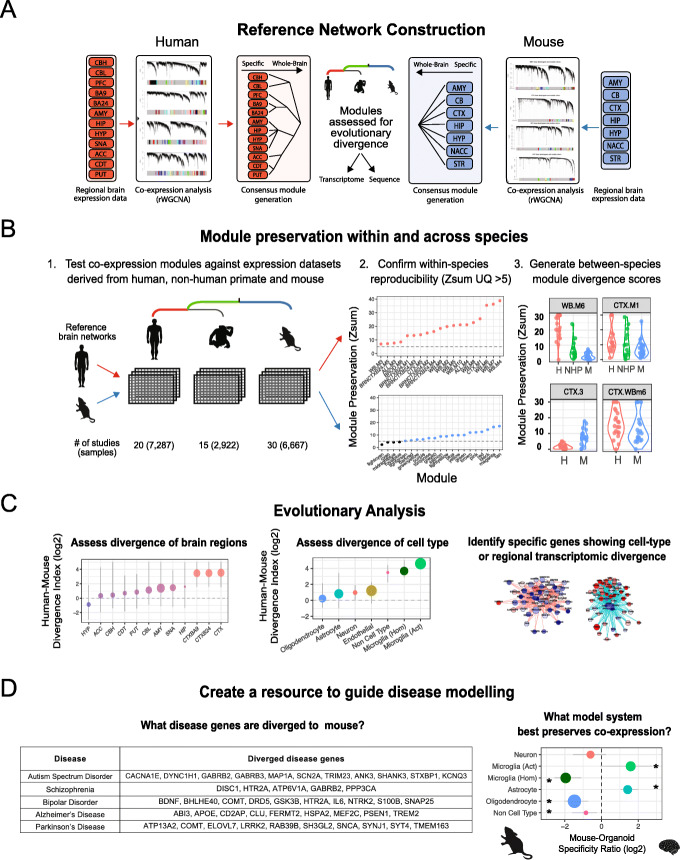
Assessing the evolutionary divergence of human and mouse brain networks. **a** Generation of human and mouse brain networks. RNA-seq expression data derived from 12 brain regions from the GTEx dataset and 7 brain regions from publicly available mouse data were used to create gene networks in each brain region using rWGCNA. These networks were hierarchically merged to generate a consensus whole-brain network. Co-expression modules were generated for each network and assessed for evolutionary divergence. Each module was assessed for transcriptome (also referred as “co-expression” or “module”) divergence as well as sequence divergence. **b** For each module in each brain region of the human and mouse brain reference networks, we assessed module reproducibility (preservation) in multiple independent test datasets derived from the same region in human, non-human primate (NHP), and mouse. For each test, a composite Zsummary (Zsum) statistic representing preservation was generated. For both human, NHP, and mouse, the upper quartile (UQ) of all test Zsum scores was calculated to generate a final preservation score in their respective species (hZsum, pZsum, mZsum). Modules with a same-species Zsum UQ > 5 are considered to be robust and reproducible. To assess divergence between human and mouse, a module divergence score was calculated as follows: (hZsum/mZsum) − 1. This framework was also applied using list of genes generated through alternate methods to assess how their co-expression structure has changed across evolution. **c** We subsequently used this framework generating evolutionary divergence scores to assess the transcriptomic divergence of brain regions and cell types and determine the genes which underlie this divergence. **d** Using these data, we create a resource to guide disease modeling. For example, we assess which human disease genes’ co-expression is not preserved in mouse and assess to what extent different model systems capture human co-expression patterns

We generated regional co-expression networks in a discovery dataset for both human and mouse to identify modules of highly co-expressed groups of genes (Fig. 1a; “Methods and materials”). Networks were created using a robust bootstrap-resampling approach to ensure modules were not driven by outlier samples [25, 26]. To validate whether these modules represent generalizable co-expression relationships, we performed module preservation analysis against multiple independent expression datasets derived from human (3–20 studies, depending on region; 7287 total samples), non-human primate (NHP) (5–15 studies; 2933 total samples), and mouse (6–28 studies; 6667 total samples; “Methods and materials”) to assess co-expression conservation (Fig. 1b; Additional files 2,3: Table S1,2). We determined quantitative module-level co-expression differences between species (“Methods and materials,” Fig. 1b) to assess the divergence of different brain regions, cell types, and the genes which underlie this divergence (Fig. 1b, c). We reasoned that modules highly conserved in multiple independent data sets from one species (e.g., human), but not in the independent data sets from another, were divergent between the species (“Methods and materials”). Together, we utilize these data to create a resource to guide disease modeling (Fig. 1d).

### Co-expression analysis in human and mouse reveals asymmetric transcriptome divergence across brain regions

**Fig. 2:**
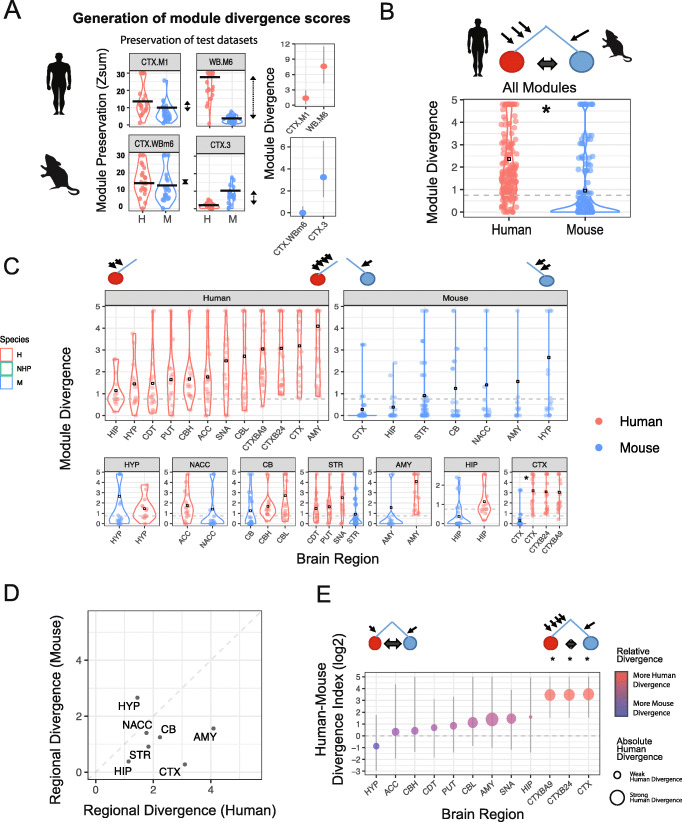
Co-expression analysis in human and mouse reveals asymmetric transcriptome divergence, greatest in the human cerebral cortex. **a** Generation of module divergence scores. Plots showing preservation (Zsum) of co-expression modules (upper—human, lower—mouse) in a number of test expression datasets derived from human and mouse (red is human, blue is mouse). Black lines represent the upper quartile (UQ) of the Zsum scores. Module divergence is calculated by comparing the UQ of the human Zsum scores vs the mouse Zsum scores. The module divergence score is rounded up to zero in those cases where the module shows greater preservation in the opposing species. For each species, we have an example of a cortical derived module that shows species-specific divergence and one which does not. Error bars represent the 95% CI from permuting across study in that species. **b** Module divergence scores for all human and mouse-derived modules, each depicted as a single dot. Human modules show significant (*; *p* < 0.05) and over twice the average (black box) transcriptomic divergence than mouse suggesting that differences between human and mouse brain are mostly due to divergence on the human lineage. **c** Divergence scores for human- and mouse-derived modules stratified by region and ordered by regional: *absolute* divergence (average module divergence; upper) and *relative* divergence (divergence index score; the ratio of the average human- and mouse-module divergence scores for each region; lower). Black box represents mean regional divergence score. Amygdala (AMY) shows greatest *absolute* transcriptomic divergence with cortical regions showing greatest *relative* transcriptomic divergence. * denotes significant (*p* < 0.05) difference between the species for regional divergence scores. **d** Regional divergence (average module divergence for each region) plotted for both human and mouse. **e** Human brain regions plotted against their relative transcriptomic divergence (Divergence Index) with point size reflecting *absolute* transcriptome divergence. Human cortical regions show the greatest *relative* and the most significant transcriptomic divergence. Error bars represent the 95% CI from permuting across study in that species; * denotes regions with a CI that does not overlap zero

To capture divergence in co-expression relationships between the species, we derived divergence scores for human and mouse modules. Module divergence scores were calculated by comparing the module preservation (Zsum) scores from test datasets of the same species (e.g., human) to the Zsum scores of test datasets derived from the opposing species (e.g., mouse) (Fig. 2a). Human modules, on average, displayed over twice the divergence than modules defined in mouse (“Methods and materials”; OR = 2.5, *p* < 1e^− 6^), suggesting an “asymmetric” transcriptomic divergence, with more changes occurring on the human lineage (Fig. 2b). The preservation of most mouse modules in human suggests that core transcriptional programs in mouse are shared with human. However, since many human modules show divergence from mouse, there may be biological processes in human with additional levels of transcriptomic complexity that are not captured in mouse.

We explored this notion of asymmetric divergence further by stratifying module divergence according to brain region, allowing us to assess regional divergence in co-expression. We observe that the cerebral cortical regions show the greatest asymmetric divergence; human-derived, but not mouse-derived, cortical modules were more diverged in the opposing species (*p* < 1e^− 3^; Fig. 2c, e). On the other hand, the cerebellum shows similarly minimal divergence for both human and mouse, suggesting this region has not strongly diverged for either species (Fig. 2c–e). The amygdala and hypothalamus both show substantial divergence (> 1.4 average module divergence) in *both* human and mouse, meaning that there are several modules that are divergent in each region in each species. While these regions possess a small degree of asymmetric divergence, this does not reach significance (Fig. 2c–e). We confirmed that this regional level trend of module divergence was not confounded by sample size, or the number of studies contributing to that network (Additional file 1: Fig. S1A). Another potential confounding factor would be the variation in the proportion of different cell-type modules across the brain regions. We tested this by normalizing regional divergence, so that the proportion of different cell-type modules were equal across regions, and found that regional divergence was not driven by the regional proportion of cell-type modules (Additional file 1: Fig. S1B).

### Glial cell types display the greatest transcriptomic divergence in human

**Fig. 3:**
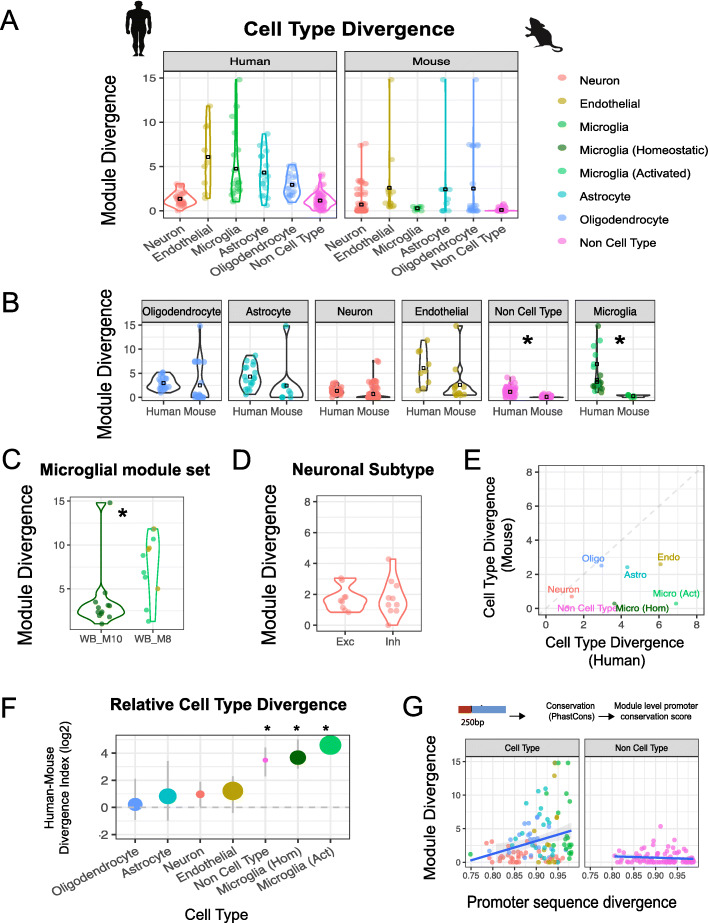
Characterizing the relationship of cell-type and promoter sequence to transcriptomic divergence. **a, b** Divergence of co-expression modules (left—human-derived, right—mouse-derived, bottom—combined) stratified by cell type. Black box represents mean divergence score for each cell type and * represents a significant difference in cell-type divergence between the species. Glial enriched modules display the greatest divergence in human and mouse, with the exception of microglial modules which show strong divergence in human but not mouse. The cell-type color scheme applies to all panels of the figure—the microglial subclassification only applies from panel **b** onwards. **c** Module divergence scores of human modules with a microglial module set name. Microglial modules fall into two main classes—WB.M10 is more representative of homeostatic microglial markers, whereas WB.M8 is more representative of microglial activation. WB.M8 displays significantly (*; *p* < 0.05) stronger divergence than WB.M10. Dot color represents module cell-type annotation. **d** Module divergence scores of human modules with enrichment for either excitatory (Exc) or inhibitory (Inh) neuronal markers. There is no difference in divergence between the two subclasses. **e** Cell-type transcriptomic divergence is plotted for both human and mouse lineages. **f** Divergence Index (ratio of mean human- and mouse-module divergence scores) plotted for major cell types with point size reflecting *absolute* transcriptome divergence. Microglia display the greatest relative divergence. Error bars represent the 95% CI from permuting across study in that species. **g** Module divergence plotted against module-level sequence divergence. Promoter conservation was assessed by measuring sequence conservation of the 250 bp upstream of each gene using the mammalian PhastCons score. The conservation score for every gene in a module was averaged to produce a module sequence divergence score. Transcriptomic divergence significantly correlates (*p* < 0.01; cor = 0.27) with promoter sequence divergence across cell types but not for modules without cell-type enrichment (cor = − 0.04; pval = 1)

To further understand which biological processes were preserved or diverged across species, we tested modules for their enrichment in cell-type marker gene lists and gene ontology terms (“Methods and materials”; Additional file 1: Fig. S1C-D, S2A-B). The cell-type classification of each human module was highly associated with human-mouse module divergence (*p* < 1e^− 10^; Kruskal Wallis), with glial-classified modules significantly more divergent than neuronal (OR > 3.1; *p* < 1e^− 6^; pairwise *t*-test; Fig. 3a, b). Human microglial modules show the largest divergence (mean = 4.8), followed by astrocytes (4.3), oligodendrocytes (2.9), and neurons (1.4). In contrast, modules with no evidence of cell-type enrichment were generally well preserved in mouse (mean = 1.1). This category of highly conserved, non-cell-type-specific modules included those enriched for GO terms relating to ribosomal-related processes, RNA binding, and energy production (Additional file 1: Fig. S2C).

To confirm that the difference in divergence between different cell types is not related to network construction, we performed preservation analysis using collated cell-type marker gene lists derived from co-expression and single-cell experiments (“Methods and materials”; Additional file 1: Fig. S2A) and found a strong correlation with our cell-type module divergence scores (Additional file 1: Fig. S2D; Pearson’s cor = 0.69 (co-expression) and 0.88 (single-cell)). To assess whether species cell-type differences may arise from differential aging trajectories [27, 28], we tested module preservation in young adult (human 13–40 years; mouse 2–14 months) and middle age to aging adult (human > 40 years; mouse > 14 months) samples (Additional file 1: Fig. S2E; “Methods and materials”). Preservation scores in human are similar between young and aging adult brain indicating that co-expression modules are not driven by age-dependent processes (Additional file 1: Fig. S2E-F). Preservation was also largely similar between aging states in mouse (Additional file 1: Fig. S2E-F). Notable exceptions were human microglial modules WB.M8 and WB.M10, which displayed stronger preservation in aging mice than young mice (OR > 1.8; pval<1e ^− 10^; Additional file 1: Fig. S2F), and are described in more detail below.

Human microglial marker genes largely fall into two module subclasses, WB.M8 and WB.M10. These modules are generated utilizing all 12 brain regions and thus are referred to as whole-brain (WB) modules (Fig. 3c). To provide more refined annotations of these modules, we assessed module enrichment using microglial marker gene lists derived from immunopanned microglia, and brain tissue from both homeostatic and pathological conditions ([29–32]; “Methods and materials”). Based on this, M10 represents a more canonical microglial signature defined by strong enrichment in canonical homeostatic microglial signatures (Additional file 1: Fig. S2G), whereas M8 represents a more activated microglial state, as defined by enrichment of endothelial genes and microglial genes upregulated in pathological conditions, such as Nfkbia and Cxcl1 ( [31]; Additional file 1: Fig. S2G). Differences between these two states explain much of the variation observed for microglial module divergence, with the activated microglial modules (WB.M8) displaying stronger divergence between species (OR = 4; *p* < 0.01; Fig. 3c). We also observe that there is a range of preservation of these microglia modules in mice, with relatively higher preservation in older versus young adult mice (Additional file 1: Fig. S2E-F). Nevertheless, the preservation in older mice is still much less than preservation in independent human datasets (human to mouse preservation OR = 4.0, M8; OR = 2.0, M10), indicating that a large proportion of microglial differences are consistent across age.

Given that microglial state appears to influence module divergence, we also sought to determine whether the two major neuronal subclasses, excitatory and inhibitory neurons display differences in their divergence. We identified modules representing inhibitory or excitatory neurons via enrichment of neuronal markers ([33]; “Methods and materials”) and compared the module divergence between these two sets (Fig. 3d). We observed no significant differences between these subclasses (*p* = 0.78). We also attempted to directly test the difference in divergence between these cell types by performing a supervised module preservation approach (“Methods and materials”) using inhibitory and excitatory neuronal markers derived from single-cell analysis [33]. Again, there was no evidence for differences in divergence between these two neuronal subgroups (Additional file 1: Fig. S2A), so we performed downstream analysis considering neurons as a general cell class.

Compared with neurons, glial cell classes displayed strong asymmetric divergence in addition to showing the greatest absolute divergence from human to mouse. Human-derived microglial modules, on average, showed the most such divergence: over ten-fold greater divergence to mouse than mouse-derived microglial modules to human (Fig. 3b, e, f). Stratification of modules by cell type in each brain region revealed that in cerebral cortex, all cell types displayed significant asymmetric divergence, i.e. human-derived modules from all cell types were significantly more diverged to mouse than their corresponding mouse-derived cell-type modules were to human (Additional file 1: Fig. S2H). In contrast, most cell types across other brain regions did not show significant asymmetric divergence (Additional file 1: Fig. S2H).

### Transcriptomic divergence is highly associated with regulatory sequence divergence for cell-type modules

The divergence of co-expression from human to mouse could be driven by many factors, genetic or environmental. Matching environmental variables between the two species is not experimentally feasible; so to understand potential mechanisms underlying divergence, we focused on the extent to which genetic variation could explain the differences in co-expression observed between species. We reasoned that we could assess the relationship of co-expression divergence to the divergence in relevant DNA sequences directly by comparing core regulatory sequence divergence to module divergence. We correlated the average PhastCons score for a number of definitions of the most proximal promoter region (250b, 2 kb upstream of all the genes in a module) with the module divergence score. Indeed, we observe a significant correlation (*p* < 0.01, Pearson’s cor = 0.27) between transcriptome and sequence divergence (Fig. 3g) with neuronal modules displaying greater evolutionary constraint than glial modules, in line with recent findings [34]. This transcriptome-sequence divergence correlation is maintained across a range of gene boundary parameters as a basis for sequence comparisons (Additional file 1: Fig. S3A-B), demonstrating that a cell-type-specific selection pressure is not being driven by our parameter selection. Finally, we find that this correlation of regulatory sequence-transcriptome divergence is restricted to cell-type-specific modules, rather than the more general metabolic, or housekeeping annotated modules, as the correlation between sequence and expression divergence is lost when assessing modules without cell-type enrichment (Fig. 3g).

### Co-expression preservation is highly associated with coding sequence constraint in cell-type modules

We next asked, given the positive relationship between module divergence and regulatory sequence divergence, whether other measures of selective pressure, such as protein coding constraint in humans was related to module divergence. Genes vary in their tolerance to loss of function (LoF) mutations on one allele [35] and can be classified depending on their frequency of non-inherited, spontaneously arising (de novo) mutations in the human population [24]. For genes where LoF mutations are well tolerated, the observed number of de novo mutations equals the expected number of mutations under the null model. Conversely, haploinsufficient genes, where half the total level of a gene product is insufficient for organismal survival, the observed de novo mutation frequency is defined to be < 10% than what is expected under the null model [35]. The pLI (probability of LoF Intolerance) score is the probability that a given gene is haploinsufficient and therefore intolerant to LoF variation and under purifying selection. We asked whether haploinsufficiency was evenly distributed across modules or related to the degree of co-expression divergence, finding a significant negative relationship between the degree of transcriptomic divergence and module enrichment for LoF intolerant genes (pLI ≥ 0.9; Pearson’s cor = − 0.2; *p* < 0.01; Additional file 1: Fig. S3C; [24]). Modules with limited transcriptomic divergence, such as neurons, manifested the greatest enrichment in genes intolerant to LoF mutations. Since functionality of these genes is necessary for organismal survival, we would expect these genes to be under tighter transcriptional regulation. Conversely, glial modules are under-represented for LoF-intolerant genes, which is consistent with the relaxed constraint of these genes at the sequence and transcriptional level.

Another measure of coding sequence constraint that reflects cross-species divergence, rather than within human constraint, is the dN/dS ratio [36–38]. For a particular gene or coding sequence, this measures the number of mutations that affect the amino acid sequence (non-silent/non-synonymous; dN) versus the number mutations that do not (silent/synonymous; dS). A gene under strong selective constraint will have many silent mutations, but very few that induce protein changes and will therefore have a dN/dS score close to zero. Genes under no selective constraint will have a dN/dS score of ~ 1, whereas genes that undergo positive selection may have a dN/dS > 1. We found a significant positive correlation between dN/dS and module divergence (Pearson’s cor = 0.35; *p* < 0.01; Additional file 1: Fig. S3D), which again indicates relaxed constraint of these genes at both the sequence and transcriptional level. These two different analyses of coding sequence constraint, coupled with the regulatory sequence analysis above, further supports the functional significance of module divergence by demonstrating its relationship to independent measures of selection and constraint at the DNA sequence level.

### Identification of modules displaying accelerated divergence after the last common ancestor (LCA) with non-human primates

**Fig. 4:**
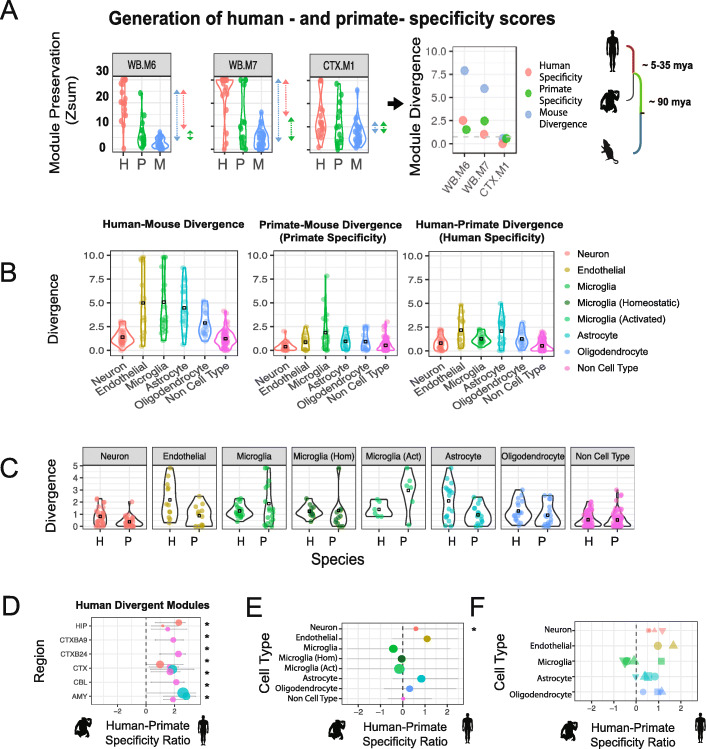
Assessing phylogenetic origins of human divergence. **a** Preservation (Zsum) scores of three human co-expression modules in test expression datasets derived from human, non-human primate (NHP), and mouse. While human-mouse-module divergence is calculated by comparing the upper quartile (UQ) of the human Zsum scores against the mouse Zsum scores, this score can be further partitioned into human and primate specificity scores by considering the NHP UQ Zsum score. This subdivision allows us to determine these transcriptomic differences arose. **b** Human-mouse divergence, human specificity, and primate specificity scores for cell-type modules in human. Black box represents mean divergence/specificity score for each cell type. The cell-type color scheme applies to all panels of the figure—the microglial subclassification only applies from panel **c** onwards. **c** Human specificity and primate specificity scores for all cell-type modules in human, grouped by cell type. Black box represents mean specificity score for each cell type. **d** Summary plot showing modules with significantly increased human specificity. Module placement on the *X*-axis is based on its human-primate specificity ratio; modules to the left of the dashed line show greater primate specificity, whereas modules to the right show greater human specificity. Points are sized by their absolute divergence to mouse. Error bars represent the 95% CI from permuting across study in that species; * denotes regions with a CI that does not overlap zero. **e** Summary plot showing mean human-primate specificity ratio for each cell type calculated by averaging the module ratio scores for each cell-type class. Most cell types trend towards human specificity. Points are sized by their absolute divergence to mouse. Error bars represent the 95% CI from permuting across study in that species; * denotes regions with a CI that does not overlap zero. **f** Summary plot showing mean human-primate specificity ratio for each cell type, having derived cell-type markers from independent (“Methods and materials”; Additional file 12: Table S11) co-expression (square; Kelley at al., 2018), single-cell RNA sequencing (triangle up; Lake et al., 2018), and cell-sorting-based experiments (triangle down; Zhang et al., 2016), which are in general highly concordant. Points are sized by their absolute divergence to mouse. Trends observed for GTEx-derived modules (**e**) are mirrored in these additional datasets

Understanding transcriptome divergence between human and mouse is essential due to the ubiquitous nature of mice in biomedical research [2]. However, it is also of interest to know if certain diseases are caused by disruption of biological processes changing most during human evolution, or whether they can be modeled in non-human primates (NHP), or human stem cell systems [12, 39]. Comparison of the Zsum scores for human, NHP and mouse allowed the Human-Mouse divergence score to be partitioned into a (1) “human-specific” component, where transcriptional changes occurred on the human lineage after divergence with the last common ancestor (LCA) of NHP, and a (2) “primate-specific” component, where changes occurred before divergence with the LCA of NHP (Fig. 4a–c).

To quantify whether module divergence was greater before or after the LCA with primates, we compared the human and primate specificity scores to each other, producing a Human-Primate “Specificity Ratio” (Fig. 4d; “Methods and materials”). If the preservation scores in NHP are closer to the preservation scores in mouse relative to human, it suggests more changes have occurred *after* the LCA of NHP, on the human-specific lineage. Conversely, if the preservation scores in NHP are closer to the preservation scores in human relative to mouse, it suggests more changes have occurred *before* the LCA of NHP, on the primate lineage. We were able to identify 13 co-expression modules where the NHP preservation scores were significantly closer to mouse relative to human and therefore displayed stronger divergence on the human-specific rather than primate lineage (Fig. 4d). We were unable to identify any modules where the NHP module preservation scores were significantly closer to human than mouse, which would have been indicative of increased divergence on the primate lineage before the NHP-human divergence.

By grouping together modules from the same cell type, and assessing preservation in NHP relative to human and mouse, we can determine the extent to which certain cell types have diverged before or after the LCA of NHP. In general, cell-type (neuronal, glial, endothelial) modules showed slightly greater trends towards transcriptional changes on the “human-specific” portion of the lineage tree compared with modules that did not have a clear cell-type identity. However, as the variability was higher among the non-neuronal cell types, the neuronal cell class was the only that reached statistical significance for human specificity (*p* < 0.05; Fig. 4b–e). This human-favored specificity ratio trend was also observed when preservation analysis was performed using cell-type marker gene lists derived from three different approaches: co-expression, cell sorting to obtain purified cell populations, and single-cell-based methods (Fig. 4f; “Methods and materials”).

### Specific genes showing cell-type or regional transcriptomic divergence

**Fig. 5:**
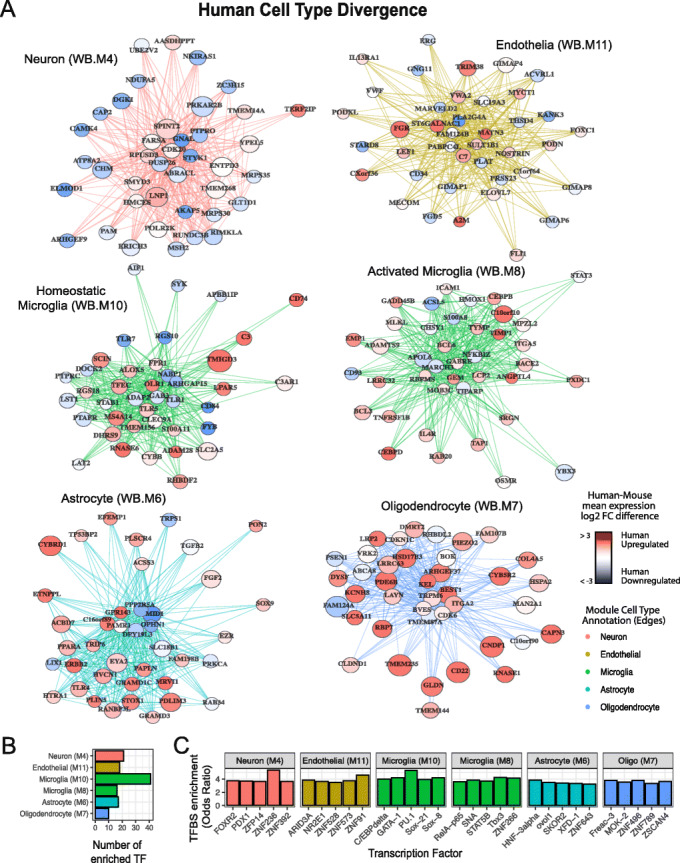
Assessing divergence and candidate regulatory drivers of cell-type modules. **a** Network plots of the significant top 40 most divergent genes for each consensus (WB) cell-type module. The top 750 connections ranked by weighted topological overlap for the consensus network are shown as node edges. Nodes with larger number of connections appear more central in each network plot. Node size relates to kME divergence. Node color relates to mean expression fold-change between human and mouse (red—upregulated in human, blue—downregulated in human). Diverged genes are available in Table S3. **b** Bar plot showing the number of TFs with binding site enrichment (OR > 3; *p* < 0.05) in promoters of diverged genes for each consensus cell-type module. **c** Bar plot showing the top five TFs whose binding sites are enriched in promoters of diverged genes for each consensus cell-type module

To identify gene drivers of module divergence, we calculated the correlation of each gene to its module eigengene (kME) in the discovery dataset and all test datasets in human, NHP and mouse. The difference in mean kME values between human and mouse was used to calculate a kME divergence score, which we utilized to highlight genes whose expression is highly divergent between human and mouse (“Methods and materials”). To highlight genes underlying cell-type divergence, we calculated the kME divergence for each gene in the consensus “whole-brain” modules across all brain regions. “Whole-brain” consensus modules were created from a co-expression network generated by creating a consensus of all regional co-expression networks (“Methods and materials”) and therefore represent shared features of co-expression across the brain. Overall, we identified 2217 genes that displayed consensus divergence across all 12 brain regions in human and 528 genes that displayed consensus divergence across all 7 brain regions in mouse (Additional files 4, 5: Table S3, S4). For human, we identified hundreds of genes whose co-expression is highly divergent for microglia (295; WB.M8/WB.M10), astrocytes (281; WB.M6), oligodendrocytes (272; WB.M7), neurons (469; WB.M4), and endothelia (88; WB.M11) (all listed in Additional file 4: Table S3). These include 1109 cell-type-associated genes that have not been previously determined to be divergent between mouse and human [8, 19], such as *TMIGD3, C3AR1* (microglia; WB.M10), *MID1, PDLIM3* (astrocyte; WB.M6), *CD22, FAM124A* (oligodendrocyte; WB.M7), *JAKMIP1*, and *NDUFA5* (neuron; WB.M4; Additional file 4: Table S3). To illustrate human-mouse cell-type divergence, network plots display the top 40 most divergent genes for each consensus cell-type module (Fig. 5a). As expected, highly divergent modules possessed a greater proportion of divergent genes (Pearson’s cor =0.48, *p* < 1e^− 11^; Additional file 1: Fig. S4A). Supporting our methodology, we find that previously identified human- and mouse-specific cell-type markers display significantly stronger kME divergence than background (Additional file 1: Fig. S4B; Human; OR = 4.1; *p* < 1e^− 13^; Mouse; OR = 14.5; *p* < 0.01 [19]; Human; OR = 2.2; *p* < 1e^− 4^ [8]).

To further assess the gene regulatory mechanisms of co-expression divergence for these cell-type modules, we sought to assess whether binding sites for particular transcription factors (TF) were enriched in the promoters of diverged genes for each cell-type module (“Methods and materials”). In total, we identify 123 TFs whose binding sites were enriched in the promoters of these diverged cell-type genes (Fig. 5b, Additional file 6: Table S5). For each cell-type module, we plot the top five (by enrichment odds ratio) of these TFs, which can be viewed as candidates for contributing to these cell type-specific changes in co-expression between human and mouse (Fig. 5c). To further assess whether a change in co-expression or expression of the TF itself may be driving the downstream effects, we sought to assess whether these implicated TFs had a higher kME divergence or expression difference than background. We found that these TFs do not show significantly greater “correlation divergence” (FC = 1.37, *p* = 0.33), but may be trending for showing relatively fewer “expression differences” (FC = 0.76, *p* = 0.09). This suggests that in general, Human-Mouse cell-type transcriptomic divergence observed here is not driven by evolutionary changes in expression and co-expression of TFs (trans effects). Instead, as suggested by the analysis showing a positive relationship between module divergence and promoter sequence divergence (Fig. 3g), divergence appears to be more driven by cis-regulatory effects.

To highlight human-specific gene associations for each cell type, we compared gene-gene correlations in all human and mouse datasets. We selected the top 100 gene pairs from each “whole-brain” consensus module (“Methods and materials”) that showed the strongest correlation differences between human and mouse (Additional file 7: Table S6) [8, 19]. As expected these genes show strong kME divergence (mean kME div = 0.17; OR = 2.3; *p* < 1e^− 16^) with the strongest effect seen in glial modules (Additional file 1: Fig. S4C). For example, in oligodendrocyte module WB.M7, the recently identified Alzheimer’s risk gene HSPA2 [40] shows 7 times stronger human-specific association with a number of oligodendrocyte markers in human than in mouse (Additional file 1: Fig. S4D), in line with a previous study [8]. For microglial module WB.M10, in addition to C3, which has previously been identified as a human-specific microglial marker [19], we show that the complement cascade genes C1QA-C and C3AR1 form 44 of the top 100 highly divergent gene pairs. This strongly implicates a human-specific role for complement-mediated synaptic pruning in microglia. Glutamate transporters SLC1A3 and SLC1A2 (EAAT1/EAAT2) show strong divergence with other members of the astrocytic WB.M6 module suggesting *human* astrocytes have an increased capability to provide glutamate to adjacent neurons.

**Fig. 6:**
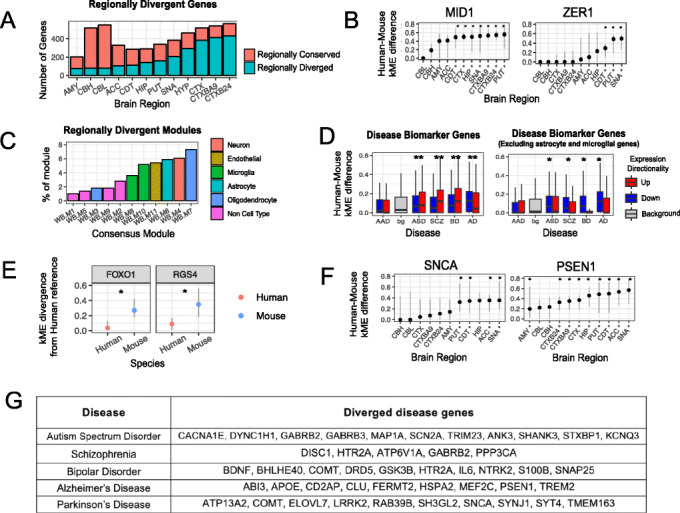
Assessing genes for regional divergence and disease association. **a** Region-specific divergent genes (blue) across all brain regions. Red bars represent genes with conserved co-expression in that region but divergent in another. The cerebral cortex displayed the greatest region-specific divergence whereas cerebellum displayed strongest region-specific conservation. **b** kME divergence of MID1 and ZER1 for each brain region. Each gene shows divergence in one region which significantly exceeds that from another region. Error bars represent the 95% CI from permuting across study in that species; * denotes divergent regions with a CI which does not overlap the CI of another region. **c** Proportion of each whole-brain consensus modules with region-specific divergent genes. Bar color indicates cell-type annotation of module. **d** The top 500 up- and downregulated genes for alcoholism (AAD), autism (ASD), bipolar disorder (BD), schizophrenia (SZ), and Alzheimer’s (AD) are plotted for their kME divergence and tested in enrichment for diverged genes (left). All disorders except for AAD show enrichment for diverged genes. Removing genes associated to an astrocytic (WB-M6) or microglial (WB-M8/WB-M10) module ablated the upregulated signature for ASD, SCZ, BD, and AD (right). **e** kME divergence of FOXO1 and RGS4 from the discovery GTEx dataset to both human and mouse datasets. FOXO1 and RGS4 are up- and downregulated respectively in SZ, BD, ASD, and AD and both display significantly increased kME divergence in mouse. Error bars represent the 95% CI from permuting across study in that species. **f** kME divergence of PSEN-1 and SNCA across all brain regions. Error bars represent the 95% CI from permuting across study in that species; * denotes regions with a CI that does not overlap zero. **g** Examples of genes diverged in at least one brain region and associated with major diseases and disorders investigated in this analysis

We also identify 1135 region-specific divergent genes, where kME divergence is restricted to particular brain regions (Additional file 8: Table S7). The cerebral cortex displayed the greatest region-specific divergence with over 300 genes significantly more diverged than at least one other brain region (Fig. 6a). For example, MID1, a member of the astrocytic WB.M6 module shows significantly stronger kME divergence in cerebral cortical regions than cerebellum (Fig. 6b). The cerebellum displayed strong region-specific conservation (Fig. 6a). This strong regional conservation is likely a combination of the general conservation of cerebellum from human to mouse (Fig. 2e) and the large transcriptional differences of the cerebellum to the rest of the brain [15, 23, 41]. Furthermore, genes comprising the neuronal (WB.M4) and oligodendrocyte (WB.M7) modules show the greatest region-specific divergence, with 6.1% and 7.3% of genes within each module respectively, displaying significantly greater divergence in a particular brain region (Fig. 6c). This suggests that neurons and oligodendrocytes manifest the most divergence between the species on a region-specific basis.

We sought to test the hypothesis that human divergent genes would have greater expression in humans, relative to mouse. We assessed whether kME diverged genes were associated with changes in mean expression between human and mouse, finding that genes with greater expression in human (> 4 FC) show greater divergence (OR = 1.4; pval< 1e^− 8^; Additional file 1: Fig. S4E). For example, highly diverged genes in the oligodendrocyte (WB.M7) and astrocyte (WB.M6) module appear to be strongly upregulated in human compared with mouse (Fig. 5a, Additional file 1: S4E), suggesting that upregulation of gene expression is one mechanism driving higher co-expression.

To assess whether this change in expression levels between species is due to a change in gene regulation, or simply a change in cell proportion, we compared human-mouse expression differences from our cortical bulk expression data to single-cell expression data derived from human and mouse cortex [7]. Genes with species differences in bulk data that maintain differences in single-cell expression data are likely to be driven by species differences in gene regulation, whereas genes with species differences only in bulk data may be driven by differences in cell-type proportion. For cell types captured in both human and mouse single-cell datasets, there is a significant (*p* < 0.01) positive correlation between bulk- and single-cell data for the species differences in gene expression, with the line of best fit passing close to the origin (Additional file 1: Fig. S4F). This indicates that cell-type proportion is not a major driver of these expression differences, but rather expression differences are largely due to cellular gene expression differences. We illustrate the single-cell expression data for four genes with greater expression in human for both bulk and single-cell expression data, indicating intracellular upregulation in human for a particular cell type (Additional file 1: Fig. S4G). Divergent neuronal genes (WB.M4) generally displayed downregulation in bulk expression data, which was mirrored in single-cell data, indicative of an intracellular downregulation of neuronal genes in human (Additional file 1: Fig. S4F, H). This matches known age-dependent repression of neuronal genes on the primate lineage [28].

Genes with divergent co-expression from human to mouse were more likely to show upregulation in both human single-cell and human bulk expression datasets. Nevertheless, a large portion (73%) of significantly divergent genes showed similar or lower expression in human (Additional file 1: Fig. S4E), indicating that changes in baseline expression level is not the sole mechanism. In addition, many genes that displayed strong expression differences between human and mouse in both single-cell and bulk datasets, did not show a strong divergence of co-expression (Additional file 1: Fig. S4E). This trend was especially true for downregulated genes—15% of human-upregulated genes (> 2 FC) in both single-cell and bulk data did not display significant divergence in co-expression, whereas 66% of human-downregulated genes (< 0.5 FC) did not show significant co-expression divergence. This suggests that species differences in mean expression can partially predict species differences in co-expression, but there is a notable proportion of genes where this trend does not apply, further indicating that differential expression is only one mechanism driving differential co-expression [42, 43].

### Gene drivers of transcriptomic divergence in disease

Mouse models are widely used to understand the molecular and cellular mechanisms underlying human disease [2]. Therefore, we reasoned that understanding which disease-associated genes were conserved for co-expression between human and mouse would help inform disease modeling. Genes up- and downregulated in human cortex from schizophrenia (SZ), bipolar disorder (BD), autism (ASD), and Alzheimer’s (AD), but not alcoholism (AAD) are significantly enriched for diverged genes (*p* < 0.01; Fig. 6d) [44]. For example, FOXO1 and RGS4 are up- and downregulated respectively in the cerebral cortex of patients with SZ, BD, ASD, or AD [45], but their co-expression is highly diverged in mouse (Fig. 6e). The divergent co-expression of these genes between species suggests they contribute to different biological processes in mice and humans [8, 19], potentially limiting their study or use as disease biomarkers in mouse. We next assessed whether the divergence of these disease-related expression changes was related to specific cell types or biological process. Indeed, genes upregulated in ASD, SCZ, BD, and AD largely represent transcriptomically divergent glial and immunological signatures, as enrichment of kME divergent genes in upregulated genes for these disorders is attenuated when omitting genes from astrocyte or microglial annotated modules (Fig. 6d).

Many transcriptional changes in post-mortem tissue may be a consequence of disease rather than causal, prompting us to focus on genes with evidence of causality in human disease (“Methods and materials”). We first examined enrichment of high-confidence ASD risk genes (*n* = 136), defined by harboring high-risk, likely protein-disrupting mutations [46–48] of which SHANK3, SCN2A, and 56 other genes displayed significant kME divergence in at least one brain region (Additional file 9: Table S8). Notably, when we extend the analysis to include the co-expression module representing synaptic vesicle trafficking in which SHANK3 is a core member (BRNHIP.M5; kME = 0.91; 28th percentile), we find that 27 out of the 200 genes in that module, including SRF, DOC2B, and LRP3 (Additional file 11: Table S10) also display significant kME divergence between human and mouse, providing further evidence that some of the basic biological pathways in which SHANK3 participates are also divergent. In total, 70 ASD risk genes were divergent when also including genes with relatively high levels of statistical support (within SFARI categories 1 or 2; *n* = 170 [49]).

Of the genes with an association to a neurodegenerative disease (*n* = 84), 40 displayed significant kME divergence from mouse (Additional file 9: Table S8). For example, alpha-synuclein (SNCA), a Parkinson’s (PD) risk gene, shows co-expression divergence between human and mouse primarily in the substantia nigra and basal ganglia, whereas presenilin-1 (PSEN-1), an AD risk gene, which had been shown to be divergent in cortex [8] displayed significant divergence across 10 brain regions (Fig. 6f; Additional file 9: Table S8). We supplement these gene lists by assessing the divergence of genes within relevant KEGG disease pathways or the DisGeNet database and provide these as a resource to guide disease modeling in mouse (Fig. 6g; “Methods and materials”; Additional file 10: Table S9).

Leveraging NHP expression data, we were able to identify 1670 genes with human-specific co-expression changes (strong kME divergence in human with respect to both NHP and mouse) such as the astrocyte gene, ACBD7, and the neuronal, PD risk gene SYT4 (Additional files 9,10: Table S8,9). One thousand six hundred and sixty genes displayed primate-specific co-expression changes (strong kME divergence from human and NHP to mouse; Additional file 11: Table S10) of which 23 had high-confidence disease association. This provides guidance as to genes that would be more aptly modeled in NHPs than mouse and suggests that modeling these disease genes in mouse should be approached with caution when attempting to relate mechanisms identified in mouse to primates or humans. These genes include the ASD risk genes SCN2A and SHANK3, the PD risk gene COMT and the AD risk gene PSEN-1 (Additional file 9: Table S8), which has previously been shown to be divergent in its co-expression between human and mouse cerebral cortex [8].

### Identifying modules strongly preserved in human cortical spheroid models

Recent advances in in vitro modeling offer the potential to model human brain development and function in a dish [50–53]. To better understand the fidelity of in vitro modeling for specific cell types, we assessed cerebral cortex module divergence for canonical cell-type modules in human cortical organoids derived from 8 separate studies [51–59]. We then compared module divergence scores between mouse, NHP, and organoids to assess which species and systems can recapitulate human neurobiology. Species “divergence scores” largely represent transcriptomic differences shaped by evolution, whereas organoid “divergence scores” reflect differences between in vivo and in vitro systems. Nevertheless, these scores can still be used to assess the applicability of using particular species or systems to model human in vivo signatures.

**Fig. 7:**
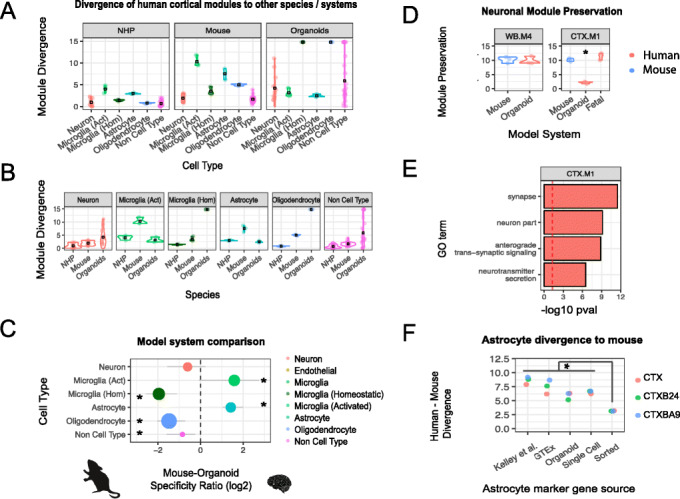
Human astrocytes are strongly preserved in human cortical organoid models. **a, b** Human cortex module divergence scores are assigned to a cell type and tested for preservation against NHP, mouse, and cortical organoid models. Black box represents mean divergence score for each cell type. Divergence scores are faceted by **a** model system and **b** cell type. **c** Summary plot of the log2 fold-change ratio between mouse divergence and brain organoid divergence for each cell type. Cell types more preserved in organoids (higher module divergence in mouse) sit to the right of the dashed line, whereas cell types more preserved in mouse (higher divergence in organoids) sit to the left of the dashed line. The size of the point represents the divergence of that cell type in the system which recapitulates the cell type most appropriately. A small dot (e.g., neuron) indicates that cell type is well captured in at least one of the two systems, whereas a large dot (e.g., oligodendrocyte) indicates that neither system models that cell type appropriately. Error bars represent the 95% CI from permuting across study in that species; * denotes cell types with a CI that does not overlap zero. **d** Preservation of cortical modules WB.M4 and CTX.M1 derived from CTX, CTXB24, and CTXBA9 tested in mouse and organoid datasets. WB.M4 shows preservation in both systems, whereas CTX.M1 is only preserved in mouse. CTX.M1 is also preserved in human fetal brain suggesting that this process is not limited to late neurodevelopmental stages. **e** Functional annotation of cortical module CTX.M1 that is preserved in mouse but not organoid. **f** Dot plot showing module divergence of astrocyte markers derived from GTEx co-expression (WB-M6), co-expression (Kelley et al., top 200 genes), organoid (top 200 genes with greatest FC difference in astrocytes versus neurons in day > 100 organoids), and sorted astrocytes (500 genes with significantly greater expression than other sorted cell types). Astrocyte markers derived from immunopanning experiments were devoid of human-specific co-expression patterns and displayed significantly lower divergence than all other marker sets

In general, astrocytic, activated glial, and most neuronal modules were well captured in cortical organoids. Conversely, homeostatic microglial and oligodendrocyte modules were not (Fig. 7a–c), which is expected given their absence from most organoid cultures [52, 53]. As previously shown, all cell types were generally well preserved in NHP datasets (Fig. 7a–c). Mouse data recapitulated modules with neuronal or no cell-type annotation, but displayed strong divergence for glial cell types (Fig. 7a–c).

To summarize whether in vivo mouse or in vitro human approaches more appropriately model in vivo adult human cell types, we calculated the log fold-change between the mouse and organoid module divergence scores. Modules relating to astrocytes displayed significantly greater preservation in human organoid cultures (Fig. 7b,c), whereas modules relating to homeostatic microglia and oligodendrocytes displayed significantly greater preservation in mouse (Fig. 7b,c), which is not surprising since most of these published IPSC models do not include these cell types [60]. Neuronal modules, which were generally well preserved in mouse, displayed different degrees of co-expression preservation in organoids. A canonical cross neuronal-subtype module, WB.M4, shows strong preservation in organoids (Fig. 7d). In contrast, the cortical module CTX.M1, which displays enrichment for excitatory pyramidal cell genes and ontology terms relating to synaptic transmission and vesicle transport (Fig. 7e), displayed weak preservation in organoids despite showing preservation in mid-gestational developing human brain samples (Fig. 7d; [41, 61]). Therefore, although basic neuronal cell types are present in vitro, mature synaptic transmission pathways are not, most likely due to their immature state, as previously shown in 2D cultures up to 12 weeks in vitro [62, 63]. It is clear that model systems need to be improved, as none of the mouse or organoid data analyzed here indicated that they faithfully recapitulate human oligodendrocyte and microglia transcriptional signatures (Fig. 7b), consistent with recent studies focused on comparing organoid development to in vivo human datasets [53, 57].

To confirm that organoids possess human-specific expression signatures not preserved in mouse, we assessed co-expression preservation in human and mouse using human astrocyte markers derived from organoid [51], and from co-expression (this study and [19]), single-cell [33], and immunopanned human brain [30]. Human organoid-derived astrocytes were highly divergent to mouse, similar to that of human astrocyte markers derived from co-expression or single-cell sequencing (Fig. 7f). Interestingly, astrocyte markers derived from immunopanned human brain were largely lacking in human-specific co-expression patterns and displayed divergence scores significantly lower than the other astrocyte marker gene lists (OR = 0.54, *p* < 0.05). This suggests that the human-specific component of astrocytes may be highly dependent on its physiological environment [64], as taking astrocytes out of their 3D environment reduces their divergence to mouse. As human-derived astrocytes appear to lose human-specific expression signatures after immunopanning and culturing, previously observed species-specific differences using this methodology may have underestimated true in vivo divergence [29, 30, 65].

## Discussion

Several previous studies have used gene networks to identify genes whose co-expression has human-specific features [8, 12, 18], usually focusing on a single or small number of brain regions due to data availability. Here, we perform the first comprehensive multi-region, multi-species comparison of the evolutionary divergence of co-expression networks generated from human brain derived from over 100 individuals [23]. Conservation of co-expression was tested in over 15,000 samples from 116 studies derived from human, NHP, and mouse (Additional file 3: Table S2). Comparison of transcriptomic conservation on both a brain-wide and a region by region basis, allowed the identification of biological processes and genes that have diverged in their co-expression relationships over evolutionary time. The preservation of most mouse modules in humans, and many human modules in mouse overall, suggests a basal level of co-expression structure shared between human and mouse, which is consistent with expectations and previous studies [8, 38, 66, 67]. However, the strong divergence of several specific human modules from mouse supports acquisition of transcriptomic complexity on the human lineage that is not shared in mouse. Further, module divergence is significantly related to independent measures of selection, including divergence of regulatory and protein coding constraint, indicating that it is capturing evolutionarily relevant genomic sequence characteristics.

The stronger divergence of human modules to mouse than mouse modules to human underlies our observation of “asymmetric divergence” in co-expression relationships. At a cell-type level, this asymmetric divergence is greatest for microglial modules, whereas at a regional level, this divergence is greatest in cerebral cortex, an observation consistent with known evolutionary hierarchies [12, 18]. We use this robustly defined network structure to identify a number of disease-associated genes, including PSEN-1, which was previously shown to be divergent based on an independent analysis of microarray data [8]. Combined with the current analysis, this strongly suggests that mouse models of PSEN-1 will not model human processes with high fidelity; notably mice harboring dominant highly PS-1 mutations do not show frank neurodegeneration or model human AD [68]. Other disease genes that show lack of conservation of human co-expression relationships in mouse include dozens of known ASD risk genes, including SCN2A and SHANK3. Divergent genes tended to be expressed at higher levels in human relative to mouse. We observe that this is not broadly due to changes in cell-type composition, but rather generally reflects cellular regulatory effects.

### Assessing evolutionary divergence using co-expression networks

In our analysis, we use module divergence (a ratio of two species Zsum scores) as a proxy for evolutionary divergence. This metric allows us to overcome the effect of module size on preservation (Additional file 1: Fig. S1D) and provides a more quantitative basis to compare transcriptome divergence between different processes. Assessing module preservation in non-human primate (NHP) allows prediction of whether transcriptional differences between human and mouse originated before the LCA with NHP, or reflect differences that occurred after the LCA with NHP and are therefore more human specific [69, 70]. We identify 13 modules where the NHP preservation scores were significantly closer to mouse than human, implicating greater divergence on the human, rather than primate lineage. These modules may therefore contribute to the differentiation between human and NHP brain. In the future, when additional expression datasets are generated from different NHPs, one should be able to create NHP subgroups and further refine when these expression differences were acquired.

Traditional phylogenetic methods utilize mean gene expression derived from all species of interest and use distance-based methods to construct an evolutionary tree [71, 72]. These methods assess the similarity of gene expression between species and construct a phylogeny to minimize the differences in expression according to their distance in the hierarchy. Assessing preservation of co-expression across different species allows us to explicitly assess evolutionary divergence of different biological processes and when on the phylogenetic lineage transcriptomic differences were acquired. Co-expression approaches assess the relationship of each gene to a module eigengene that is generated in a particular species. Therefore, it would be problematic to apply a distance-based approach between NHP and mouse when using values derived from a *human* co-expression network.

By constructing networks in each species and region individually, we can define the biological processes and cell types for each species independently. Other studies have combined expression datasets from different species or regions into a comprehensive expression matrix before constructing a co-expression network [12, 16]. These studies can highlight species-specific processes as co-expression modules are largely driven by genes that display differential expression between species. But, these approaches do not query species differences in co-expression, which may be unrelated to differential expression and are therefore complementary to the approach taken within our study.

The construction of regional networks yields modules relating to different cell types across regions, which permitted analysis of preservation in co-expression across difference species in cell subtypes. Single-cell sequencing has enabled the detection of numerous cell classes in both human and mouse, permitting identification of species differences at the cell-type level. Whereas cell-type matching between species allows identification of differentially expressed genes between species [7], expression levels themselves may obey a largely neutral model [17]. Because co-expression reflects functional mechanisms such as co-regulation, changes in network position reflect changes in function [18, 21]. In this regard, we observe that human-upregulated genes tend to display stronger kME divergence, consistent with potential adaptive evolution. Still, a substantial portion (73%) of kME divergent genes show similar or reduced expression levels in humans (Additional file 1: Fig. S4E), the latter of which might be interpreted as more consistent with a model of neutral evolution [17]. For example, the astrocytic gene, PARD3B, shows stable interspecies expression levels (< 0.5 logFC) in both bulk and single-cell expression data, but shows strong human to mouse divergence at the co-expression level (kME div = 0.51; *p* < 0.01), which indicates a functional change. On the other hand, IL17D shows considerably higher expression (> 2 logFC) in human bulk and single-cell data, yet is not significantly divergent for co-expression, consistent with a neutral model. Differential expression has been successful in identifying gene expression differences between cell types or brain regions; however, preservation of gene co-expression has been suggested to be more successful in recapitulating evolutionary hierarchies [18, 70] and may therefore be more suited to assessing functional differences between species.

Single-cell sequencing may not detect genes with low levels of expression, which generally reside in the periphery of cell-type co-expression modules (Additional file 1: Fig. S5A-B). As genes on the periphery of cell-type modules show the greatest divergence of co-expression (Additional file 1: Fig. S5C-D), until a greater depth can be afforded, co-expression analysis of bulk tissue sequencing will remain important for identifying evolutionary differences, as co-expression analysis of bulk expression data captures genes across a wider range of expression and network position, not just the most central [73].

An important recent study used co-expression analysis to identify “high-fidelity” markers for broad cell classes across a number of brain regions in both human and mouse [19]. Although our identified diverged human-mouse genes are highly overlapping with species specific “high-fidelity” markers [19], our study approached this issue of divergence differently, starting with a discovery dataset, and subsequently assessing co-expression in independent test datasets from different species. Differences in preservation between studies may be due to technical differences, such as RNA-extraction method and sequencing platform, or biological differences such as subject age and housing conditions. Identifying evolutionary differences unrelated to these study differences will therefore increase the signal associated with true evolutionary differences between species. We bootstrapped the effect of study to create confidence intervals around all module and gene divergence scores, allowing us to assess the potential impact of these technical and biological “study” effects.

### Modeling brain function and disease in mouse

Given the ubiquitous nature of mouse in biomedical research for modeling neurological disease [2], it is important to understand species-specific differences. We observe that human glia are highly diverged from mouse suggesting that it may be difficult to make extrapolations of these cell types in human, especially when considering the transcriptome. For example, many transcriptomic perturbations in neuropsychiatric diseases (Fig. 6d; [44, 74]) are associated with immune-glial activation, a response likely to be symptomatic of neuronal dysregulation. Therefore, the initial neurobiological causes of these diseases may be recapitulated in mouse models, but their downstream transcriptional outputs may differ.

By calculating gene-level divergence, we highlight genes that may drive the divergence of cell types and other biological processes in human. For example, ACBD7 and CYBRD1, both in the astrocytic module WB.M6, have both been highlighted in a recent paper to be human-specific astrocyte markers [19], which we confirm in our analysis (kMEdiv ≥ 0.4; *p* < 0.01). Kelley et al. also showed that PMP2, another previously identified human-specific astrocyte gene, when upregulated in mouse astrocytes, was able to increase the number of primary processes and size of mouse astrocytes [19], which is a well-known distinction between human and mouse [6]. In our dataset, of all genes, PMP2 displayed the greatest change in expression between human and mouse and showed strong divergence of co-expression in CTX (kMEdiv = 0.54; *p* < 0.01). As supported by single-cell data, PMP2 is associated with both astrocytes (WB.M6; mean kME = 0.48) and oligodendrocytes (WB.M7, mean kME = 0.29), which may have prevented module assignment in other regions [7]. In addition to these specified genes, we identify hundreds of significantly diverged genes for astrocytes and other cell types, for which functional experiments may elucidate the genes effect on making their respective cell type in mouse more “human” (Additional file 4: Table S3). For example, as the functional effect of PMP2 upregulation was relatively modest [19], we predict upregulating additional genes may allow human and mouse cell types to become increasingly comparable.

Assessing the top 100 divergent gene pairs from each “whole-brain” consensus module can highlight novel human-specific functions for each cell type (Additional file 7: Table S6). Human-specific associations of glutamate transporters SLC1A3 and SLC1A2 (EAAT1/EAAT2) in the astrocytic WB.M6 module suggest *human* astrocytes have an increased capability to provide glutamate to adjacent neurons. Numerous highly divergent genes of the oligodendrocyte WB.M7 module (e.g., PSEN-1, HSPA2) are associated with Alzheimer’s (AD) [40, 75]. Furthermore, strong divergence of WB.M7 gene pairs involved in carnosine (CARNS1, CNDP1) and copper (SLC31A2) metabolism suggests a human-specific role for metal homeostasis in oligodendrocytes. Additionally, the AD risk gene TREM2 [76] resides among the highly divergent microglial WB.M10 genes. The complement cascade genes C1QA-C, C3, and C3AR1 also form many of the highly divergent gene pairs within the microglial WB.M10 module, suggesting a human-specific role for complement-mediated synaptic pruning in microglia, which may have implications for both AD and ASD disease pathophysiology [77, 78].

We identify dozens of genes currently associated with risk for neurodegenerative and neurodevelopmental disorders whose co-expression is significantly diverged from mouse (Additional file 9: Table S8). Remarkably, alpha-synuclein (SNCA), a PD risk gene, shows divergence primarily in the substantia nigra—the first region to display degeneration in PD patients [79]. Presenilin-1 (PSEN-1), an AD risk gene, displayed divergence to mouse across numerous brain regions, but was preserved in NHP. PAX6 and ERLIN2, in the astrocyte module WB.M6, are both implicated in intellectual disability and display co-expression divergence across all cerebral cortex regions. SHANK3 was among 57 other ASD risk genes (“Methods and materials”; [46–48]), displaying significant kME divergence in at least one brain region and we provide a full list in Table S8. Notably, our data provide strong evidence that some of the basic biological pathways in which SHANK3 participates are also divergent, suggesting that modeling in primate or human in vitro systems is likely to more faithfully recapitulate disease pathophysiology. Together, these findings demonstrate a number of genes that contribute to human disease, but whose function is not likely to be faithfully recapitulated in mouse.

### In vitro models of human brain and cell types

Recent advances in in vitro modeling of human brain offers the potential to model human brain function in a dish [50–53]. Cortical organoids faithfully recapitulated astrocytic, activated glial, and most neuronal in vivo co-expression signatures. Although oligodendrocyte and homeostatic microglial signatures were not captured, future analyses should attempt to incorporate these cell types appropriately [60, 80]. Currently, given that aging mouse brain most successfully recapitulated the microglial co-expression signature, some microglial related processes may be more appropriate to study in aging mice. But, once microglia are faithfully incorporated into 3D organoid models, their preservation should be carefully tested [80, 81]. The most notable advantage of cortical organoids compared to mouse is the faithful recapitulation of human astrocytes, which appear to model human astrocytes similarly to NHP in vivo. For example, ARHGEF6, a member of the astrocytic WB.M6 module is associated with X-linked mental retardation and is significantly more preserved in organoids than mouse, making organoids a preferred model to study mechanisms underlying this gene’s role in disease.

Interestingly, astrocyte (and oligodendrocyte) markers derived from sorting experiments [30] did not show strong divergence in co-expression from human to mouse (Additional file 1: Fig. S2D). These cell types were sorted based upon HepaCam and GalC markers respectively and therefore may not have captured all glial sub-populations, some of which perhaps representing more highly diverged, human-specific aspects of glial biology. Alternatively, immunopanning and culturing of human astrocytes may remove them from their physiologically optimal state in a 3D environment and cause them to lose their human-specific properties, both transcriptionally and functionally. Interestingly, mice with brain-engrafted human glial progenitors and astrocytes displayed enhancement of both activity-dependent plasticity and learning [82]. So, although physiological environment may be important for astrocytes to manifest their human-specific components, this environment may be somewhat shared between human brain, cortical organoids, and mouse brain.

### Limitations and further work

This study highlights a number of transcriptomic differences between species, especially for glial cell types. Most identified differences are likely due to evolutionary differences between species; however, we cannot exclude the effect of external confounding factors such as environment, diet, or agonal state. For example, given the sterile housing conditions of mice, we hypothesize that immunological differences in human could be due to non-sterile conditions. We cannot rule out some contribution of environmental differences to the divergence of this activated glial signature. But it is important to note that regardless of cause, this cell state is not captured in mouse. Mitigating against a major or pervasive contribution of environmental effects to these differences, we find that co-expression divergence was strongly correlated with sequence divergence, which would drive the differential regulation of gene expression [38, 83]. Moreover, we observed that this activated microglial signature was not specific to humans, but also was observed in NHP housed in laboratory conditions.

We also focused on one-to-one ortholog relationships, which represented over 90% of the human-mouse gene relationships. This focus on one-to-one orthologs greatly simplified the interpretation of co-expression preservation between species. Although relatively small in number, genes with distinct orthologs in one or the other species are more likely to be divergent indicating that our analysis may understate the extent of transcriptomic divergence between the species [84]. Future work can assess how distinct orthologs fit within the co-expression framework defined here [85].

Additionally, to assess module preservation, we utilize the combination of many test expression datasets that were not assayed uniformly across all brain regions in this study. Therefore, module preservation of each brain region may utilize a different combination of test datasets, which may differ by sample preparation, developmental time point, or environmental state. To assess the effect of study selection upon regional divergence, we regressed out any “study-specific” effects upon module divergence and observe that regional divergence after study regression is correlated with the raw regional divergence scores (Additional file 1: Fig. S1E). This suggests the non-uniform distribution of test expression datasets across brain regions does not bias regional preservation scores, although there may still be a small confound between brain region and factors underlying study design. We perform study-level permutations to calculate region-specific divergence differences to further account for the variability in study choice to mitigate this issue.

This study provides a multi-region, multi-species comparison of the evolutionary divergence of transcriptomic networks generated from adult human brain. However, the brain also exists under a number of different developmental states or environmental conditions, which would need to be further investigated to achieve a more complete understanding of species differences. However, these analyses, based on dozens of data sets, and multiple brain regions, provide a robust framework for understanding major species differences.

## Methods and materials

### Acquisition of expression datasets

To assess the evolutionary conservation of brain networks, we processed 7287 samples from 12 brain regions in human—cerebellum (CBL), cerebellar hemisphere (CBH), dorso-lateral pre-frontal cortex (CTX), Brodmann area 9 (CTXBA9), Brodmann area 24 (CTXB24), hippocampus (HIP), amygdala (AMY), hypothalamus (HYP), substantia nigra (SNA), nucleus accumbens (ACC), caudate nucleus (CDT), and putamen (PUT); 2933 samples from six brain regions—cerebellum (CBL), cortex (CTX), hippocampus (HIP), amygdala (AMY), hypothalamus (HYP), and basal ganglia (BG), of three non-human primates (NHP)—macaque, baboon, and chimpanzee; and 6667 samples from seven brain regions—cerebellum (CBL), cortex (CTX), hippocampus (HIP), amygdala (AMY), hypothalamus (HYP), nucleus accumbens (NACC), and striatum (STR), in mouse (Additional file 2: Table S1). We also measured network preservation against in vitro brain organoid systems from eight independent studies (Additional file 3: Table S2). Only organoids older than day 50 were used for this analysis as a compromise between statistical power and accurate matching to mature human brain. Individual studies are listed in Supplementary Table 2. Given the lack of independent hypothalamic expression datasets in NHP, this brain region was omitted from NHP-related analysis.

### Processing of gene expression data

Unless specified, normalized expression values were obtained from GEO or from the study authors directly. For mouse RNA-seq data, samples were aligned to the GrCm38 transcriptome using Salmon (v0.7.2) producing TPM values [86]. Genes were retained if they had > 20% non-zero values for each study of a brain region and were subsequently log2 (+ 0.001) transformed. To ensure co-expression was not driven by outlying samples, a sample was removed if its connectivity (sum of the sample’s correlation to the other samples) was low (z.k (scaled connectivity) < − 2.5), or if the sample displayed over 4 SDs from the mean for any of the top 10 expression PCs. Outlier removal was iterated up to 5 times or until < 3 samples were removed in an iteration. All available technical (i.e., batch, alignment statistics) and biological covariates (i.e., age, sex, sub-region) were removed from the expression data using a regression model providing that the covariate explained on average over 1% of the expression variance, while ensuring that at least 8 degrees of freedom remained in the dataset. For the mouse RNA-seq datasets, both STAR (v.020201) and picard (v.2.5.0) covariates were log2 (+ 0.001) transformed and combined to make a set of sequencing PCs that were included as possible technical covariates for regression [87]. If the expression dataset sample size was < 10, covariates were not regressed. To allow comparison between human, NHP, and mouse datasets, gene IDs were converted to their corresponding ortholog Ensembl gene ID using the biomaRt package (v.2.35.1) in R [88].

### Network generation

To generate human and mouse brain networks, we utilized a bootstrapped-resampling version of WGCNA [26, 89] on 12 brain regions in human using RNA-seq data from the GTEx consortium [22] and 7 brain regions in mouse using a number of publicly available RNA-seq datasets (Additional file 3: Table S2). To prevent the effect of study driving mouse network construction, we removed batch effects between study using ComBat (v3.20.0) [90] before combining datasets into a single expression matrix. For each regional expression matrix, 50 signed co-expression networks (Topological Overlap Matrices (TOMs)) were generated using Pearson’s correlation from 50 independent bootstraps of the samples with each bootstrapped co-expression network using the same estimated power parameter. The power parameter selected was the smallest power (between 4 and 20) which achieves a truncated *r*^2^ over 0.8 and a negative slope. If the *r*^2^ does not reach 0.8, the power selected was 20. A regional consensus co-expression network was generated by taking the median of each edge across all 50 bootstrapped TOMs. Regional networks were then merged edge-wise in a hierarchical manner until a whole-brain (WB) consensus network was generated [23]. In mouse, the whole-brain consensus network was created using a consensus of the 7 region-specific networks, having subset for common genes.

Networks were hierarchically clustered using average linkage (using “1 – TOM” as a dis-similarity measure). Human modules were previously generated using a cut height which maximizes the correlation between bootstrap and consensus TO scores while keeping the minimum module size to 50 [23]. In mouse, modules were created by selected a deepsplit cut height (between 0 and 3) that gave the number of modules closest to 20 (as this was approximately the number of human modules generated) while setting the minimum module size to 50. We removed modules which had limited support from independent microarray datasets (Zsum UQ < 5). For each region, the nomenclature of each WB consensus module would be adopted by the regional module that shared the greatest similarity (Jaccards Index > 0.4). The “M” within each module name denotes “Module.” The remaining regional modules in mouse were numerically named according to module size, for instance AMY.1 and AMY.6 being the largest and smallest amygdala modules respectively that did not show significant similarity to a WB consensus module.

### Module annotation

For cell-type enrichment, a logistic regression test was performed for each module against cell-type marker gene lists derived from sorting experiments [29–32, 65]. The model was written as *glm (is.in.module ~ is.cell.marker, family = binomial)* which generated a *p* value and odds ratio (OR). This method was used for all enrichment analysis in this manuscript. A module was assigned to the cell type for which it had the strongest enrichment, providing the OR was above 3 and did not show stronger enrichment for mitochondrial- or ribosomal-related genes. Gene ontology was assessed using the R package gProfileR (v.0.7.0) [91].

To identify modules representative of inhibitory or excitatory neuronal cell types, we assessed the enrichment of modules for inhibitory and excitatory markers derived from single-cell analysis (Additional file 12: Table S11) [33]. If a module possessed an enrichment score > 2 for a particular neuronal subtype which was also at least 20% greater than the enrichment for the opposing neuronal subtype, it would be assigned to that corresponding neuronal subtype. This criterion was selected to provide an adequate number of modules in both neuronal subclasses.

### Calculating module preservation scores

To query the extent to which a module was reproducible (preserved), every module of each reference network was tested against many independent expression datasets derived from the same, or highly similar, brain region for each species (Additional file 2: Table S1). Preservation analysis was also performed using marker gene lists in addition to co-expression modules (Additional file 12: Table S11). For analysis comparing young and aging adult brain, samples were partitioned into two groups (young and aging adult) for each study (*n* = 6 human; *n* = 5 mouse) on a regional basis. Young adult is defined as 13–40 years old in human and 2–14 months old in mouse. Aging adult is defined as 40+ years in human and 14+ months in mouse. Module preservation was executed using 25 permutations. For each test, a composite Zsummary (Zsum) statistic was generated. The larger the *z*-score the more likely it is to be preserved in the test dataset, traditionally a Zsum score > 10 indicates strong module preservation whereas a score < 2 suggests weak preservation. Zsum scores were capped at 40 to prevent inflated aggregate preservation scores. The Zsummary score is generated from a number of other statistics such as module density (how densely connected the module genes are in the test network) or connectivity (how well connected the module genes are in the test network). For further details, see [25]. The Zsum score shows a strong dependence on module size—with larger modules having greater statistical significance to be called preserved than smaller modules (Additional file 1: Fig. S1D). However, this dependence on module size does not affect module divergence scores as these are calculated as a ratio of two preservation scores.

### Calculating module divergence scores

The upper quartile (UQ) of all Zsum preservation scores was calculated for human, non-human primate (NHP), and mouse independently, generating UQ hZsum, UQ pZsum, and UQ mZsum scores respectively. The UQ was chosen to compare between evolutionary classes in order to remove emphasis from lowly preserved datasets, perhaps collected from an unrelated developmental stage or differing expression profiling platform. Modules with a same-species UQ Zsum score of over 5 were deemed reproducible and retained for further analysis (Fig. 1b). Comparing the UQ Zsum scores between species allows us to determine module-level co-expression differences between species. To assess divergence for each human and mouse module, the fold-change (FC) difference was calculated between the UQ hZsum and UQ mZsum scores (Fig. 2a). These divergence scores were capped at a score of 15 to remove the effect of extreme outliers. There were a small portion of modules which displayed negative divergence scores but were relatively small in magnitude and were therefore rounded up to 0.01 to indicate no divergence and allow downstream calculations. These negatively divergent modules were members of highly preserved cell classes and therefore likely a result of the normal distribution in divergence observed around 0 (no divergence).

95% confidence intervals were calculated for each divergence score, by permuting across study. For each permutation, Zsum scores were sampled with replacement and an UQ score generated which was subsequently used to calculate a module divergence score. For each module, we generated and ordered 1000 permuted divergence scores. The 25th and 975th divergence score was selected as the 95% CI.

For all human modules, to gain a greater understanding of when these transcriptomic changes were acquired, we were also able to leverage the UQ pZsum scores to create a (1) “human specificity” (HS) score, where transcriptional changes occurred on the human lineage after divergence with the last common ancestor (LCA) of NHP, and a (2) “primate specificity” (PS) score, where changes occurred before divergence with the LCA of NHP (Fig. 4a). Human specificity can also be interpreted as Human-Primate divergence, whereas primate specificity interpreted as Primate (including human)-Mouse divergence. The HS score is the fold-change difference between the UQ hZsum score and the UQ mZsum or UQ pZsum score (whichever was larger). The PS score is the fold-change difference between the UQ hZsum score or UQ pZsum score (whichever is smaller) and the UQ mZsum score. These calculations can be summarized as below.

$$ \mathrm{Mouse}-\mathrm{Human}\ \mathrm{divergence}\ \left(\mathrm{Mouse}\ \mathrm{modules}\right)=\left(\mathrm{UQ}\ \mathrm{mZsum}/\mathrm{UQ}\ \mathrm{hZsum}\right)-1 $$

$$ \mathrm{Human}-\mathrm{Mouse}\ \mathrm{divergence}\ \left(\mathrm{Human}\ \mathrm{modules}\right)=\left(\mathrm{UQ}\ \mathrm{hZsum}/\mathrm{UQ}\ \mathrm{mZsum}\right)\kern0.37em -1 $$

$$ \mathrm{Human}\ \mathrm{Specificity}\ \left(\mathrm{Human}-\mathrm{Primate}\ \mathrm{Divergence}\right)\ \mathrm{score}=\left(\mathrm{UQ}\ \mathrm{hZsum}/\max \left(\mathrm{UQ}\ \mathrm{pZsum},\mathrm{UQ}\ \mathrm{mZsum}\right)\right)\kern0.37em -1 $$

$$ \mathrm{Primate}\ \mathrm{Specificity}\ \left(\mathrm{Primate}-\mathrm{Mouse}\ \mathrm{Divergence}\right)\ \mathrm{score}=\left(\min \left(\mathrm{UQ}\ \mathrm{hZsum},\mathrm{UQ}\ \mathrm{pZsum}\right)/\mathrm{UQ}\ \mathrm{mZsum}\right)-1 $$

### Calculating gene-level divergence scores

To identify gene drivers of module divergence, we calculated the correlation of each gene to its module eigengene (kME) in the discovery dataset and all test datasets in human, NHP, and mouse. Next, we calculated the kME difference between each species and the discovery dataset. The difference in mean kME values between human and mouse was used to calculate a kME divergence score, which was used to highlight highly divergent genes between species. Genes with negative kME divergence scores were rounded up to 0.01 to indicate no divergence. For each region, kME values and divergence scores were generated for both regional and whole-brain (WB) module.

### Calculating human-mouse expression differences

To assess the difference in mean expression between human and mouse, both bulk and single-cell data were used. For bulk comparison, mean TPM values were calculated for the human (GTEx) and mouse (compiled) discovery RNA-seq expression data on a region by region basis. The fold-change difference between human and mouse expression was calculated as (Human mean TPM + 0.1) / (Mouse mean TPM + 0.1). The addition of constant 0.1 was chosen as a compromise between preventing aggregated FC scores due to extremely low mean TPM values and to accurately illuminate human and mouse expression differences for lowly expressed genes. To calculate a consensus expression difference between human and mouse, TPM values were averaged across regions before calculating fold-change.

To assess the change in expression between human and mouse, while matching for cell type, we utilized single-cell expression data derived from human and mouse cortex [7]. For human and mouse respectively, we downloaded and utilized the available trimmed-mean and median expression TPM values for all genes in each of their identified cell-type clusters. For human, 120 cell-type clusters were identified, for which we assigned 110 clusters (prefixed with “Inh” or “Exc”) into a neuronal group, 3 clusters (prefixed with “Astro”) into an astrocyte group, 3 clusters (prefixed with “Oligo” or “OPC”) into an oligodendrocyte group, 1 cluster (“Micro”) into a microglial group, and 1 cluster (“Endo”) into an endothelial group. The clusters prefixed with “VLMC” and “Peri” were excluded from further analysis. For mouse, 290 cell-type clusters were identified, for which we assigned 271 clusters (variable nomenclature) into a neuronal group, 2 clusters (“Astro”) into an astrocyte group, 6 clusters (“Oligo” or “OPC”) into an oligodendrocyte group, and 3 clusters (“Endo”) into an endothelial group. The clusters prefixed with “SMC,” “VLMC,” or “Macrophage” were excluded from further analysis. To assess the expression level for each cell type, we calculated the mean expression level across all the cell-type subclusters. Given the heterogeneity of neurons and the 8:2 ratio of excitatory neurons to inhibitory neurons in the brain [92], we calculated a weighted mean for neurons, weighing the mean expression of excitatory neurons by 0.8 and the inhibitory neurons by 0.2 with the subsequent value being more representative of pooled bulk expression. To allow interspecies comparison, we converted the Mouse TPM data by subsetting genes to one-to-one human-mouse orthologs and using the human gene name equivalent.

To compare the human-mouse expression fold-change difference for each cell type, we calculated the mean expression across all clusters within a cell-type group and compared the mean expression of this cell-type between the species as such: (Human cell-type mean TPM + 0.1) / (Mouse cell-type mean TPM + 0.1). To compare the human-mouse FC difference between bulk and single-cell data, genes with co-expression membership to cell-type modules were compared with their respective cell-type in single-cell expression data.

### Disease enrichment

To assess enrichment of disease relevant genes in human-mouse diverged genes, a logistic regression test was performed for diverged genes against the top 500 genes up- and downregulated in autism, schizophrenia, bipolar disorder, depression, anxiety, and Alzheimer’s [44, 74].

To investigate genes with causal association to human disease, we curated (a) genes harboring high risk likely protein-disrupting mutations in ASD patients [46–48], (b) genes of SFARI categories 1 or 2 [49], (c) genes within a nervous system disease KEGG pathway [93], (d) genes of the DisGeNet database score linked to mental or behavioral dysfunction with an association score above 0.5 [94, 95], and (e) previously curated genes implicated in neurodegenerative diseases [96].

### Calculating sequence divergence

To measure sequence divergence of genes in each module, the 3′ UTR, 5′ UTR, promoter (250 / 2000 bp upstream of transcription start site), introns, exons, and splice sites of each gene was determined and PhastCons score generated. The PhastCons score is a measure of DNA sequence conservation across 30 different mammalian species with constrained sequences obtaining a higher score [97]. The median gene PhastCons score was calculated for each module—providing a module-level sequence divergence score for many gene annotations.

The Ensembl API (July 2019 archive) was utilized to retrieve human-mouse dN and dS scores which were subsequently used to calculate the dN/dS score.

### Transcription factor binding site enrichment

Enrichment analysis was performed using TRANSFAC(R) of the geneXplain platform. TRANSFAC(R) establishes the individual motifs enriched in the promoters of the input gene set as compared with a background set [98]. Significantly diverged genes for each whole-brain consensus cell-type module were tested for enrichment using all expressed genes as background. For each gene, the 1000 bp upstream and 100 bp downstream of the transcription start site was tested for enrichment using the “vertebrate_human_p0.001” TF profiles.

To calculate whether TFs were more greatly affected in their co-expression or expression, we compared Human-Mouse (a) kMEdiv scores and (b) absolute mean expression differences between these enriched TFs versus background (all brain expressed genes).

### Software version

R version 3.3.0 and WGCNA version 1.68 was used for the analysis described in this manuscript.

## Supplementary Information

**Additional file 1: Supplementary Figures.**

**Additional file 2: Table S1.** Total number of samples and test datasets underlying each module preservation test.

**Additional file 3: Table S2.** Reference of all expression datasets utilized in this study.

**Additional file 4: Table S3.** Consensus divergence scores for genes in human Whole Brain modules.

**Additional file 5: Table S4.** Consensus divergence scores for genes in mouse Whole Brain modules.

**Additional file 6: Table S5.** Transcription factor binding site enrichment analysis for diverged genes of consensus cell type modules.

**Additional file 7: Table S6.** Top 100 gene pair correlation divergences for human Whole Brain modules.

**Additional file 8: Table S7.** Region-specific divergent genes.

**Additional file 9: Table S8.** High confidence disease associated genes diverged from mouse.

**Additional file 10: Table S9.** Disease associated genes diverged from mouse.

**Additional file 11: Table S10.** All genes, module assignments and divergence scores.

**Additional file 12: Table S11.** Cell type marker lists used in analyses.

**Additional file 13.** Review history.

## Data Availability

The datasets supporting the conclusions of this article can be accessed through the GEO or other online repositories with unique identifiers listed in Table S2.

## References

[1] Sousa AMM, Meyer KA, Santpere G, Gulden FO, Sestan N (2017). Evolution of the human nervous system function, structure, and development. Cell..

[2] Eppig JT, Blake JA, Bult CJ, Kadin JA, Richardson JE (2015). Mouse Genome Database G. The Mouse Genome database (MGD): facilitating mouse as a model for human biology and disease. Nucleic Acids Res.

[3] Mouse Genome Sequencing C, Waterston RH, Lindblad-Toh K, Birney E, Rogers J, Abril JF (2002). Initial sequencing and comparative analysis of the mouse genome. Nature..

[4] Hedges SB, Dudley J, Kumar S (2006). TimeTree: a public knowledge-base of divergence times among organisms. Bioinformatics..

[5] Herculano-Houzel S, Mota B, Lent R (2006). Cellular scaling rules for rodent brains. Proc Natl Acad Sci U S A.

[6] Oberheim NA, Takano T, Han X, He W, Lin JH, Wang F (2009). Uniquely hominid features of adult human astrocytes. J Neurosci.

[7] Hodge RD, Bakken TE, Miller JA, Smith KA, Barkan ER, Graybuck LT (2019). Conserved cell types with divergent features in human versus mouse cortex. Nature..

[8] Miller JA, Horvath S, Geschwind DH (2010). Divergence of human and mouse brain transcriptome highlights Alzheimer disease pathways. Proc Natl Acad Sci U S A.

[9] Skene NG, Grant SG (2016). Identification of vulnerable cell types in major brain disorders using single cell transcriptomes and expression weighted cell type enrichment. Front Neurosci.

[10] Wang M, Roussos P, McKenzie A, Zhou X, Kajiwara Y, Brennand KJ (2016). Integrative network analysis of nineteen brain regions identifies molecular signatures and networks underlying selective regional vulnerability to Alzheimer's disease. Genome Med.

[11] Muchnik SK, Lorente-Galdos B, Santpere G, Sestan N (2019). Modeling the evolution of human brain development using organoids. Cell..

[12] Konopka G, Friedrich T, Davis-Turak J, Winden K, Oldham MC, Gao F (2012). Human-specific transcriptional networks in the brain. Neuron..

[13] Chimpanzee S, Analysis C (2005). Initial sequence of the chimpanzee genome and comparison with the human genome. Nature..

[14] King MC, Wilson AC (1975). Evolution at two levels in humans and chimpanzees. Science..

[15] Zhu Y, Sousa AMM, Gao T, Skarica M, Li M, Santpere G, et al. Spatiotemporal transcriptomic divergence across human and macaque brain development. Science. 2018;362(6420):eaat8077. 10.1126/science.aat8077 PMC6900982 30545855

[16] Sousa AMM, Zhu Y, Raghanti MA, Kitchen RR, Onorati M, Tebbenkamp ATN (2017). Molecular and cellular reorganization of neural circuits in the human lineage. Science..

[17] Khaitovich P, Weiss G, Lachmann M, Hellmann I, Enard W, Muetzel B (2004). A neutral model of transcriptome evolution. Plos Biol.

[18] Oldham MC, Horvath S, Geschwind DH (2006). Conservation and evolution of gene coexpression networks in human and chimpanzee brains. Proc Natl Acad Sci U S A.

[19] Kelley KW, Nakao-Inoue H, Molofsky AV, Oldham MC (2018). Variation among intact tissue samples reveals the core transcriptional features of human CNS cell classes. Nat Neurosci.

[20] Oldham MC, Konopka G, Iwamoto K, Langfelder P, Kato T, Horvath S (2008). Functional organization of the transcriptome in human brain. Nat Neurosci.

[21] Parikshak NN, Gandal MJ, Geschwind DH (2015). Systems biology and gene networks in neurodevelopmental and neurodegenerative disorders. Nat Rev Genet..

[22] Consortium GT, Laboratory DA, Coordinating Center Analysis Working G, Statistical Methods groups-Analysis Working G, Enhancing Gg, Fund NIHC (2017). Genetic effects on gene expression across human tissues. Nature..

[23] Hartl C, Ramaswami G, Pembroke W, Muller S, Pintacuda G, Saha A, et al. The architecture of brain co-expression reveals the brain-wide basis of disease susceptibility. bioRxiv. 2020:2020.03.05.965749.

[24] Samocha KE, Robinson EB, Sanders SJ, Stevens C, Sabo A, McGrath LM (2014). A framework for the interpretation of de novo mutation in human disease. Nat Genet.

[25] Langfelder P, Luo R, Oldham MC, Horvath S (2011). Is my network module preserved and reproducible?. Plos Comput Biol.

[26] Langfelder P, Horvath S. Fast R functions for robust correlations and hierarchical clustering. J Stat Softw. 2012;46(11):1-17. PMC3465711 23050260

[27] Galatro TF, Holtman IR, Lerario AM, Vainchtein ID, Brouwer N, Sola PR (2017). Transcriptomic analysis of purified human cortical microglia reveals age-associated changes. Nat Neurosci.

[28] Loerch PM, Lu T, Dakin KA, Vann JM, Isaacs A, Geula C (2008). Evolution of the aging brain transcriptome and synaptic regulation. Plos One.

[29] Zhang Y, Chen K, Sloan SA, Bennett ML, Scholze AR, O'Keeffe S (2014). An RNA-sequencing transcriptome and splicing database of glia, neurons, and vascular cells of the cerebral cortex. J Neurosci.

[30] Zhang Y, Sloan SA, Clarke LE, Caneda C, Plaza CA, Blumenthal PD (2016). Purification and characterization of progenitor and mature human astrocytes reveals transcriptional and functional differences with mouse. Neuron..

[31] Holtman IR, Noback M, Bijlsma M, Duong KN, van der Geest MA, Ketelaars PT (2015). Glia Open Access Database (GOAD): a comprehensive gene expression encyclopedia of glia cells in health and disease. Glia..

[32] Mancarci BO, Toker L, Tripathy SJ, Li B, Rocco B, Sibille E, et al. Cross-laboratory analysis of brain cell type transcriptomes with applications to interpretation of bulk tissue data. eNeuro. 2017;4(6). 10.1523/ENEURO.0212-17.2017 PMC5707795 29204516

[33] Lake BB, Chen S, Sos BC, Fan J, Kaeser GE, Yung YC (2018). Integrative single-cell analysis of transcriptional and epigenetic states in the human adult brain. Nat Biotechnol.

[34] Hu G, Li J, Wang GZ (2020). Significant evolutionary constraints on neuron cells revealed by single-cell transcriptomics. Genome Biol Evol..

[35] Lek M, Karczewski KJ, Minikel EV, Samocha KE, Banks E, Fennell T (2016). Analysis of protein-coding genetic variation in 60,706 humans. Nature..

[36] Goldman N, Yang Z (1994). A codon-based model of nucleotide substitution for protein-coding DNA sequences. Mol Biol Evol.

[37] Kimura M (1977). Preponderance of synonymous changes as evidence for the neutral theory of molecular evolution. Nature..

[38] Monaco G, van Dam S, Casal Novo Ribeiro JL, Larbi A, de Magalhaes JP (2015). A comparison of human and mouse gene co-expression networks reveals conservation and divergence at the tissue, pathway and disease levels. BMC Evol Biol.

[39] Qiu Z, Li X (2017). Non-human primate models for brain disorders - towards genetic manipulations via innovative technology. Neurosci Bull.

[40] Petyuk VA, Chang R, Ramirez-Restrepo M, Beckmann ND, Henrion MYR, Piehowski PD (2018). The human brainome: network analysis identifies HSPA2 as a novel Alzheimer’s disease target. Brain..

[41] Kang HJ, Kawasawa YI, Cheng F, Zhu Y, Xu X, Li M (2011). Spatio-temporal transcriptome of the human brain. Nature..

[42] Farahbod M, Pavlidis P (2019). Differential coexpression in human tissues and the confounding effect of mean expression levels. Bioinformatics..

[43] Amar D, Safer H, Shamir R (2013). Dissection of regulatory networks that are altered in disease via differential co-expression. Plos Comput Biol.

[44] Gandal MJ, Haney JR, Parikshak NN, Leppa V, Ramaswami G, Hartl C (2018). Shared molecular neuropathology across major psychiatric disorders parallels polygenic overlap. Science..

[45] Mirnics K, Middleton FA, Stanwood GD, Lewis DA, Levitt P (2001). Disease-specific changes in regulator of G-protein signaling 4 (RGS4) expression in schizophrenia. Mol Psychiatry.

[46] Ruzzo EK, Perez-Cano L, Jung JY, Wang LK, Kashef-Haghighi D, Hartl C (2019). Inherited and de novo genetic risk for autism impacts shared networks. Cell..

[47] Sanders SJ, He X, Willsey AJ, Ercan-Sencicek AG, Samocha KE, Cicek AE (2015). Insights into autism spectrum disorder genomic architecture and biology from 71 risk loci. Neuron..

[48] Satterstrom FK, Kosmicki JA, Wang J, Breen MS, De Rubeis S, An JY (2020). Large-scale exome sequencing study implicates both developmental and functional changes in the neurobiology of autism. Cell..

[49] Abrahams BS, Arking DE, Campbell DB, Mefford HC, Morrow EM, Weiss LA (2013). SFARI Gene 2.0: a community-driven knowledgebase for the autism spectrum disorders (ASDs). Mol Autism.

[50] Pasca AM, Sloan SA, Clarke LE, Tian Y, Makinson CD, Huber N (2015). Functional cortical neurons and astrocytes from human pluripotent stem cells in 3D culture. Nat Methods.

[51] Sloan SA, Darmanis S, Huber N, Khan TA, Birey F, Caneda C (2017). Human astrocyte maturation captured in 3D cerebral cortical spheroids derived from pluripotent stem cells. Neuron..

[52] Yoon SJ, Elahi LS, Pasca AM, Marton RM, Gordon A, Revah O (2019). Reliability of human cortical organoid generation. Nat Methods.

[53] Kanton S, Boyle MJ, He Z, Santel M, Weigert A, Sanchis-Calleja F (2019). Organoid single-cell genomic atlas uncovers human-specific features of brain development. Nature..

[54] Camp JG, Badsha F, Florio M, Kanton S, Gerber T, Wilsch-Brauninger M (2015). Human cerebral organoids recapitulate gene expression programs of fetal neocortex development. Proc Natl Acad Sci U S A.

[55] Pollen AA, Nowakowski TJ, Chen J, Retallack H, Sandoval-Espinosa C, Nicholas CR (2015). Molecular identity of human outer radial glia during cortical development. Cell..

[56] Birey F, Andersen J, Makinson CD, Islam S, Wei W, Huber N (2017). Assembly of functionally integrated human forebrain spheroids. Nature..

[57] Pollen AA, Bhaduri A, Andrews MG, Nowakowski TJ, Meyerson OS, Mostajo-Radji MA (2019). establishing cerebral organoids as models of human-specific brain evolution. Cell..

[58] Trujillo CA, Gao R, Negraes PD, Gu J, Buchanan J, Preissl S (2019). Complex oscillatory waves emerging from cortical organoids model early human brain network development. Cell Stem Cell.

[59] Velasco S, Kedaigle AJ, Simmons SK, Nash A, Rocha M, Quadrato G (2019). Individual brain organoids reproducibly form cell diversity of the human cerebral cortex. Nature..

[60] Marton RM, Miura Y, Sloan SA, Li Q, Revah O, Levy RJ (2019). Differentiation and maturation of oligodendrocytes in human three-dimensional neural cultures. Nat Neurosci.

[61] Walker RL, Ramaswami G, Hartl C, Mancuso N, Gandal MJ, de la Torre-Ubieta L (2019). Genetic control of expression and splicing in developing human brain informs disease mechanisms. Cell..

[62] Stein JL, de la Torre-Ubieta L, Tian Y, Parikshak NN, Hernandez IA, Marchetto MC (2014). A quantitative framework to evaluate modeling of cortical development by neural stem cells. Neuron..

[63] Pasca SP (2018). The rise of three-dimensional human brain cultures. Nature..

[64] Khakh BS, Deneen B (2019). The emerging nature of astrocyte diversity. Annu Rev Neurosci.

[65] Cahoy JD, Emery B, Kaushal A, Foo LC, Zamanian JL, Christopherson KS (2008). A transcriptome database for astrocytes, neurons, and oligodendrocytes: a new resource for understanding brain development and function. J Neurosci.

[66] Eidsaa M, Stubbs L, Almaas E (2017). Comparative analysis of weighted gene co-expression networks in human and mouse. Plos One.

[67] Tsaparas P, Marino-Ramirez L, Bodenreider O, Koonin EV, Jordan IK (2006). Global similarity and local divergence in human and mouse gene co-expression networks. BMC Evol Biol.

[68] Hall AM, Roberson ED (2012). Mouse models of Alzheimer’s disease. Brain Res Bull.

[69] Preuss TM, Caceres M, Oldham MC, Geschwind DH (2004). Human brain evolution: insights from microarrays. Nat Rev Genet.

[70] Varki A, Geschwind DH, Eichler EE (2008). Explaining human uniqueness: genome interactions with environment, behaviour and culture. Nat Rev Genet..

[71] Brawand D, Soumillon M, Necsulea A, Julien P, Csardi G, Harrigan P (2011). The evolution of gene expression levels in mammalian organs. Nature..

[72] Gu X, Zou Y, Huang W, Shen L, Arendsee Z, Su Z (2013). Phylogenomic distance method for analyzing transcriptome evolution based on RNA-seq data. Genome Biol Evol.

[73] Masalia RR, Bewick AJ, Burke JM (2017). Connectivity in gene coexpression networks negatively correlates with rates of molecular evolution in flowering plants. Plos One.

[74] Allen M, Carrasquillo MM, Funk C, Heavner BD, Zou F, Younkin CS (2016). Human whole genome genotype and transcriptome data for Alzheimer’s and other neurodegenerative diseases. Sci Data.

[75] Kelleher RJ, Shen J (2017). Presenilin-1 mutations and Alzheimer’s disease. Proc Natl Acad Sci U S A.

[76] Pottier C, Wallon D, Rousseau S, Rovelet-Lecrux A, Richard AC, Rollin-Sillaire A (2013). TREM2 R47H variant as a risk factor for early-onset Alzheimer’s disease. J Alzheimers Dis.

[77] Hansen DV, Hanson JE, Sheng M (2018). Microglia in Alzheimer’s disease. J Cell Biol.

[78] Hemonnot AL, Hua J, Ulmann L, Hirbec H (2019). Microglia in Alzheimer disease: well-known targets and new opportunities. Front Aging Neurosci.

[79] Surmeier DJ, Obeso JA, Halliday GM (2017). Selective neuronal vulnerability in Parkinson disease. Nat Rev Neurosci.

[80] Ormel PR, Vieira de Sa R, van Bodegraven EJ, Karst H, Harschnitz O, MAM S (2018). Microglia innately develop within cerebral organoids. Nat Commun.

[81] Abud EM, Ramirez RN, Martinez ES, Healy LM, Nguyen CHH, Newman SA (2017). iPSC-derived human microglia-like cells to study neurological diseases. Neuron..

[82] Han X, Chen M, Wang F, Windrem M, Wang S, Shanz S (2013). Forebrain engraftment by human glial progenitor cells enhances synaptic plasticity and learning in adult mice. Cell Stem Cell.

[83] Khaitovich P, Hellmann I, Enard W, Nowick K, Leinweber M, Franz H (2005). Parallel patterns of evolution in the genomes and transcriptomes of humans and chimpanzees. Science..

[84] Gabaldon T, Koonin EV (2013). Functional and evolutionary implications of gene orthology. Nat Rev Genet..

[85] Yan KK, Wang D, Rozowsky J, Zheng H, Cheng C, Gerstein M (2014). OrthoClust: an orthology-based network framework for clustering data across multiple species. Genome Biol.

[86] Patro R, Duggal G, Love MI, Irizarry RA, Kingsford C (2017). Salmon provides fast and bias-aware quantification of transcript expression. Nat Methods.

[87] Dobin A, Davis CA, Schlesinger F, Drenkow J, Zaleski C, Jha S (2013). STAR: ultrafast universal RNA-seq aligner. Bioinformatics..

[88] Durinck S, Spellman PT, Birney E, Huber W (2009). Mapping identifiers for the integration of genomic datasets with the R/Bioconductor package biomaRt. Nat Protoc.

[89] Langfelder P, Horvath S (2008). WGCNA: an R package for weighted correlation network analysis. BMC Bioinformatics.

[90] Leek JT, Johnson WE, Parker HS, Jaffe AE, Storey JD (2012). The sva package for removing batch effects and other unwanted variation in high-throughput experiments. Bioinformatics..

[91] Reimand J, Kull M, Peterson H, Hansen J, Vilo J (2007). g:Profiler--a web-based toolset for functional profiling of gene lists from large-scale experiments. Nucleic Acids Res.

[92] Trapp P, Echeveste R, Gros C (2018). E-I balance emerges naturally from continuous Hebbian learning in autonomous neural networks. Sci Rep.

[93] Kanehisa M, Sato Y (2020). KEGG mapper for inferring cellular functions from protein sequences. Protein Sci.

[94] Pinero J, Bravo A, Queralt-Rosinach N, Gutierrez-Sacristan A, Deu-Pons J, Centeno E (2017). DisGeNET: a comprehensive platform integrating information on human disease-associated genes and variants. Nucleic Acids Res.

[95] Pinero J, Ramirez-Anguita JM, Sauch-Pitarch J, Ronzano F, Centeno E, Sanz F (2020). The DisGeNET knowledge platform for disease genomics: 2019 update. Nucleic Acids Res.

[96] Fu H, Hardy J, Duff KE (2018). Selective vulnerability in neurodegenerative diseases. Nat Neurosci.

[97] Siepel A, Bejerano G, Pedersen JS, Hinrichs AS, Hou M, Rosenbloom K (2005). Evolutionarily conserved elements in vertebrate, insect, worm, and yeast genomes. Genome Res.

[98] Matys V, Kel-Margoulis OV, Fricke E, Liebich I, Land S, Barre-Dirrie A (2006). TRANSFAC and its module TRANSCompel: transcriptional gene regulation in eukaryotes. Nucleic Acids Res.

