# Supplementary material for: Evolutionary conservation and divergence of the human brain transcriptome
Source: Genome Biol. 2021 Jan 29;22:52. doi: 10.1186/s13059-020-02257-z (PMC7844938; doi:10.1186/s13059-020-02257-z)
Supplement: Supplementary file 1 — Additional file 1: Supplementary Figures. [file 13059_2020_2257_MOESM1_ESM.pdf]

Fig S1

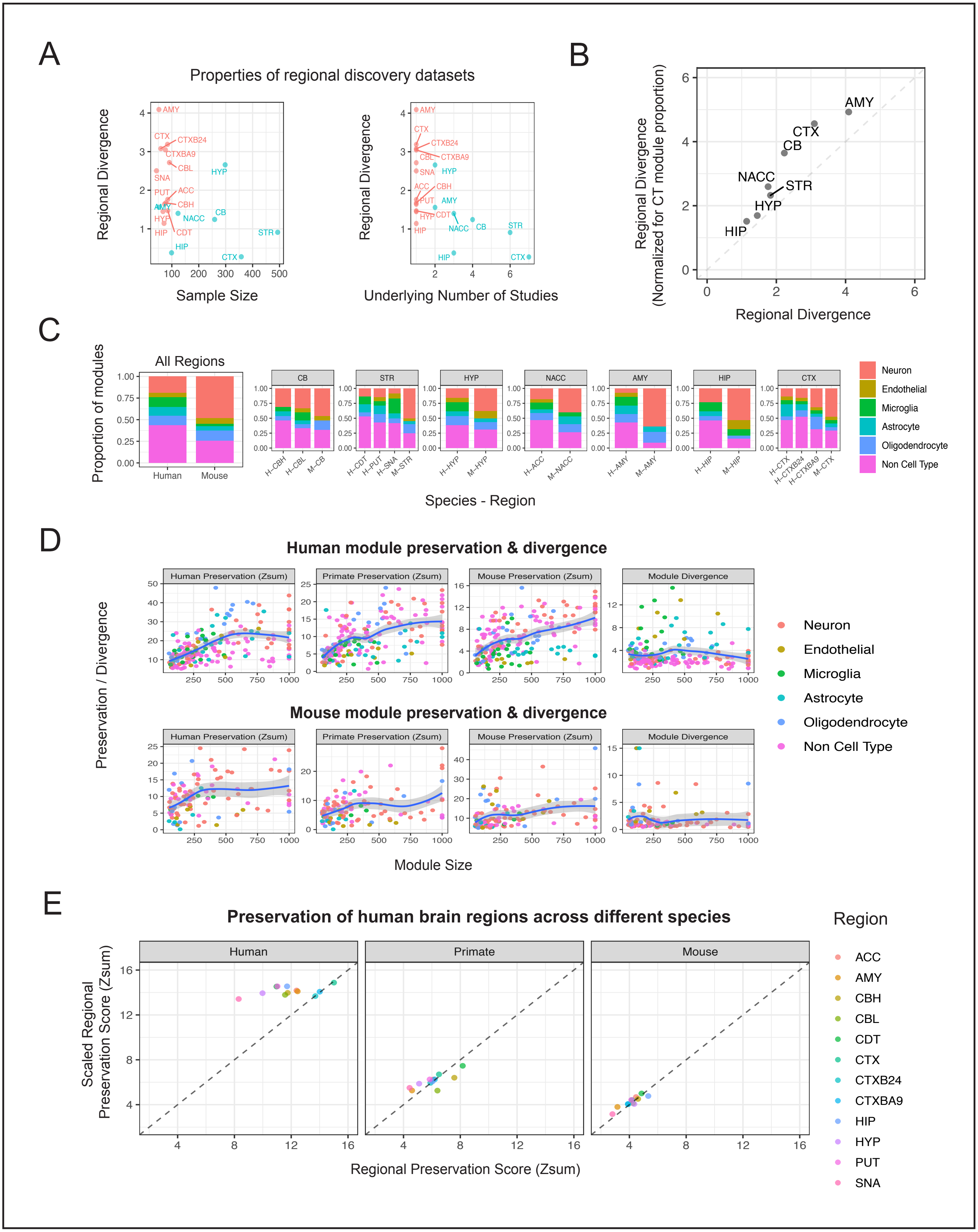
A
Properties of regional discovery datasets
Regional Divergence
Sample Size
Underlying Number of Studies
AMY
CTX
CTXB24
CTXBA9
CBL
SNA
HYP
PUT
ACC
CBH
NACC
CB
HIP
CDT
STR
B
Regional Divergence
(Normalized for CT module proportion)
Regional Divergence
C
Proportion of modules
All Regions
Human
Mouse
Species - Region
Neuron
Endothelial
Microglia
Astrocyte
Oligodendrocyte
Non Cell Type
D
Human module preservation & divergence
Mouse module preservation & divergence
Human Preservation (Zsum)
Primate Preservation (Zsum)
Mouse Preservation (Zsum)
Module Divergence
Preservation / Divergence
Module Size
E
Preservation of human brain regions across different species
Human
Primate
Mouse
Scaled Regional
Preservation Score (Zsum)
Regional Preservation Score (Zsum)
Region
ACC
AMY
CBH
CBL
CDT
CTX
CTXB24
CTXBA9
HIP
HYP
PUT
SNA

Fig S2

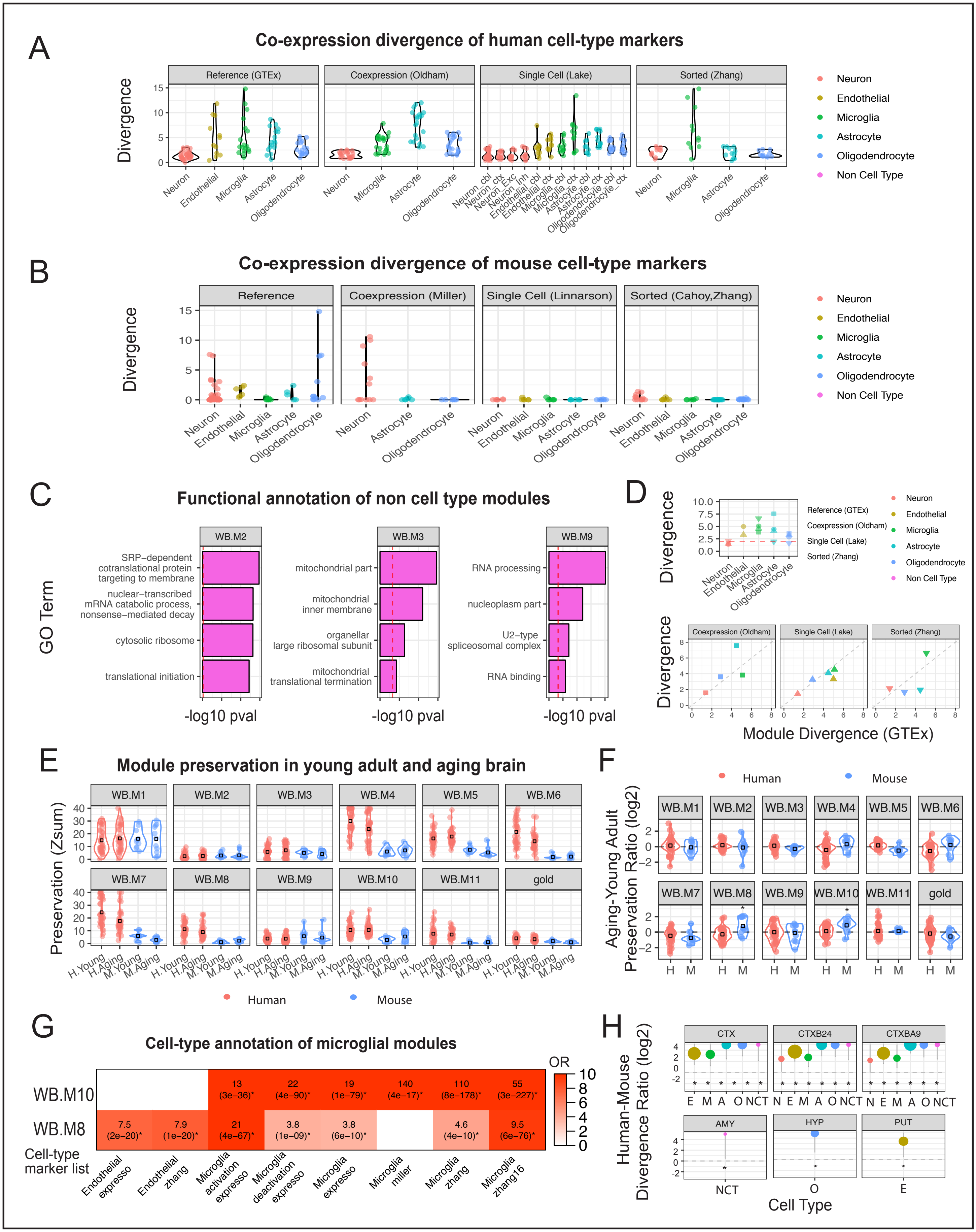
A
Co-expression divergence of human cell-type markers
Reference (GTEx)
Coexpression (Oldham)
Single Cell (Lake)
Sorted (Zhang)
Divergence
Neuron
Endothelial
Microglia
Astrocyte
Oligodendrocyte
Non Cell Type
B
Co-expression divergence of mouse cell-type markers
Reference
Coexpression (Miller)
Single Cell (Linnarson)
Sorted (Cahoy,Zhang)
C
Functional annotation of non cell type modules
GO Term
WB.M2
SRP−dependent cotranslational protein targeting to membrane
nuclear−transcribed mRNA catabolic process, nonsense−mediated decay
cytosolic ribosome
translational initiation
WB.M3
mitochondrial part
mitochondrial inner membrane
organellar large ribosomal subunit
mitochondrial translational termination
WB.M9
RNA processing
nucleoplasm part
U2−type spliceosomal complex
RNA binding
-log10 pval
D
Module Divergence (GTEx)
E
Module preservation in young adult and aging brain
Preservation (Zsum)
WB.M1
WB.M2
WB.M3
WB.M4
WB.M5
WB.M6
WB.M7
WB.M8
WB.M9
WB.M10
WB.M11
gold
H.Young
H.Aging
M.Young
M.Aging
Human
Mouse
F
Aging-Young Adult Preservation Ratio (log2)
H
M
G
Cell-type annotation of microglial modules
OR
WB.M10
WB.M8
13 (3e−36)*
22 (4e−90)*
19 (1e−79)*
140 (4e−17)*
110 (8e−178)*
55 (3e−227)*
7.5 (2e−20)*
7.9 (1e−20)*
21 (4e−67)*
3.8 (1e−09)*
3.8 (6e−10)*
4.6 (4e−10)*
9.5 (6e−76)*
Cell-type marker list
Endothelial expresso
Endothelial zhang
Microglia activation expresso
Microglia deactivation expresso
Microglia expresso
Microglia miller
Microglia zhang
Microglia zhang16
H
Human-Mouse Divergence Ratio (log2)
CTX
CTXB24
CTXBA9
AMY
HYP
PUT
E M A O NCT
N E M A O NCT
NCT
O
E
Cell Type

Fig S3

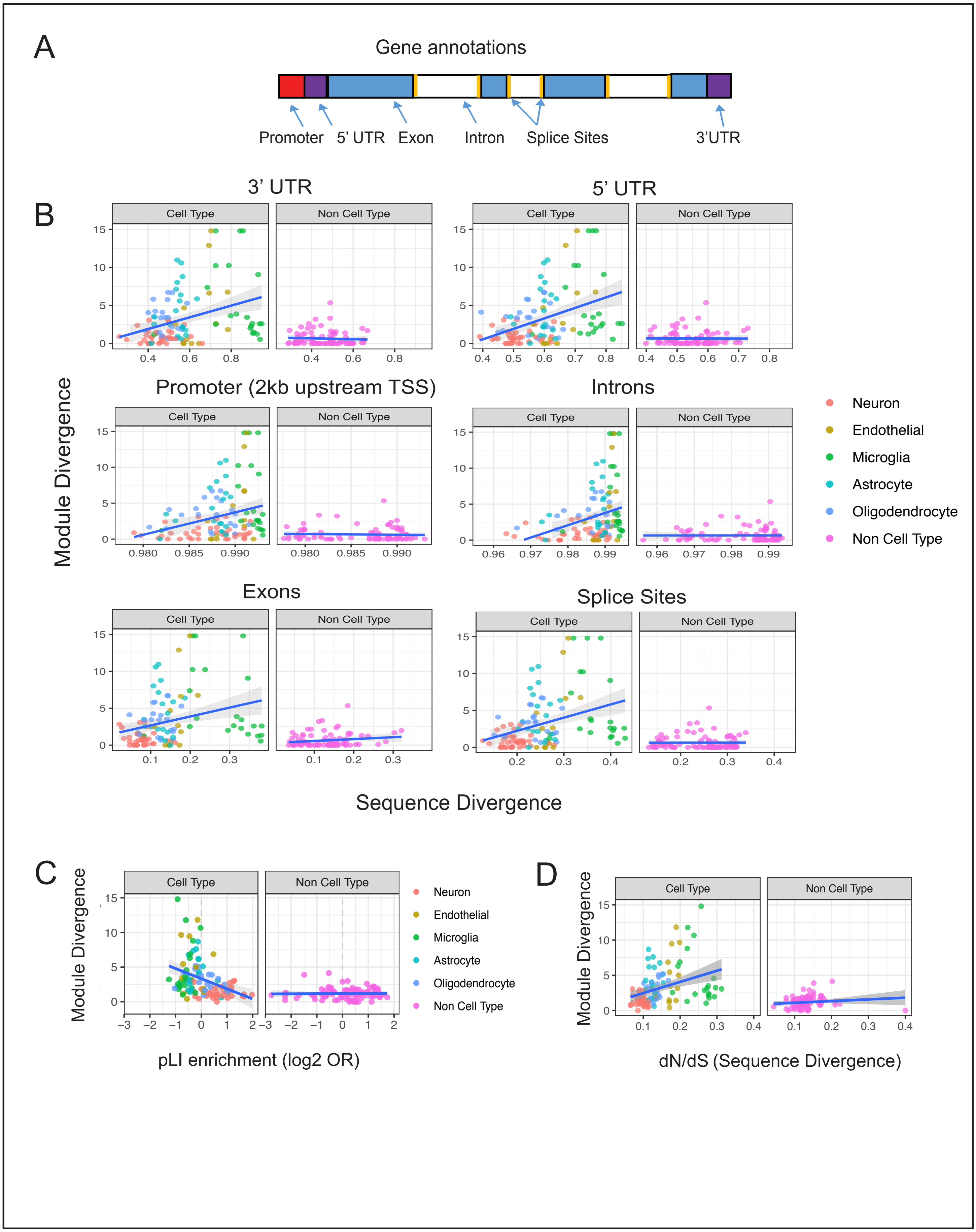

A
Gene annotations
Promoter
5' UTR
Exon
Intron
Splice Sites
3'UTR
B
3' UTR
5' UTR
Promoter (2kb upstream TSS)
Introns
Exons
Splice Sites
Cell Type
Non Cell Type
Module Divergence
Sequence Divergence
Neuron
Endothelial
Microglia
Astrocyte
Oligodendrocyte
Non Cell Type
C
Module Divergence
pLI enrichment (log2 OR)
D
Module Divergence
dN/dS (Sequence Divergence)

Fig S4

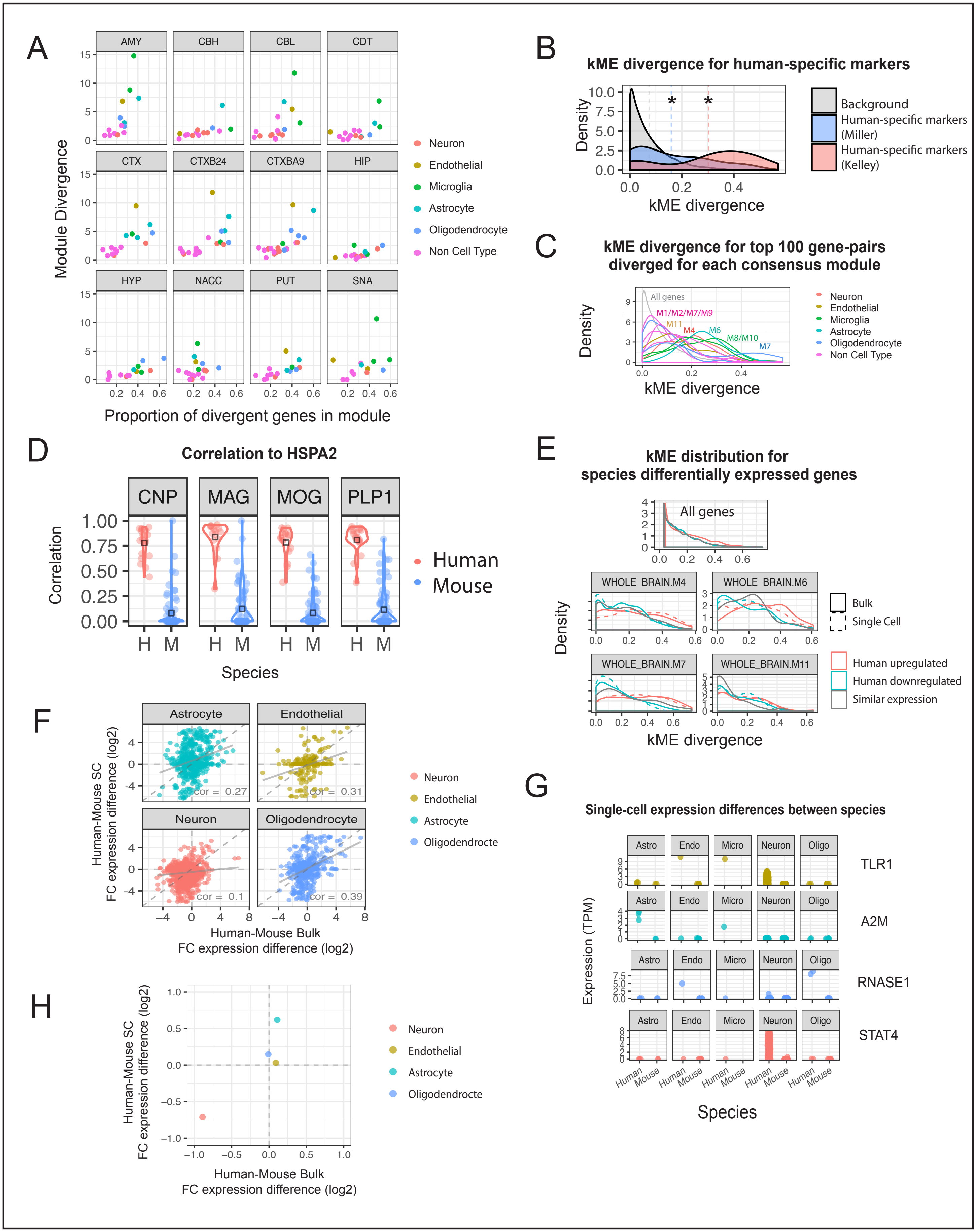

A
AMY
CBH
CBL
CDT
CTX
CTXB24
CTXBA9
HIP
HYP
NACC
PUT
SNA
Module Divergence
Proportion of divergent genes in module
Neuron
Endothelial
Microglia
Astrocyte
Oligodendrocyte
Non Cell Type
B
kME divergence for human-specific markers
Density
kME divergence
Background
Human-specific markers (Miller)
Human-specific markers (Kelley)
C
kME divergence for top 100 gene-pairs diverged for each consensus module
All genes
M1/M2/M7/M9
M11
M4
M6
M8/M10
M7
Density
kME divergence
Neuron
Endothelial
Microglia
Astrocyte
Oligodendrocyte
Non Cell Type
D
Correlation to HSPA2
CNP
MAG
MOG
PLP1
Correlation
Species
Human
Mouse
E
kME distribution for species differentially expressed genes
All genes
WHOLE_BRAIN.M4
WHOLE_BRAIN.M6
WHOLE_BRAIN.M7
WHOLE_BRAIN.M11
Density
kME divergence
Bulk
Single Cell
Human upregulated
Human downregulated
Similar expression
F
Astrocyte
Endothelial
Neuron
Oligodendrocyte
cor = 0.27
cor = 0.31
cor = 0.1
cor = 0.39
Human-Mouse SC FC expression difference (log2)
Human-Mouse Bulk FC expression difference (log2)
Neuron
Endothelial
Astrocyte
Oligodendrocte
G
Single-cell expression differences between species
Astro
Endo
Micro
Neuron
Oligo
TLR1
A2M
RNASE1
STAT4
Expression (TPM)
Human
Mouse
Species
H
Human-Mouse SC FC expression difference (log2)
Human-Mouse Bulk FC expression difference (log2)
Neuron
Endothelial
Astrocyte
Oligodendrocte

Fig S5

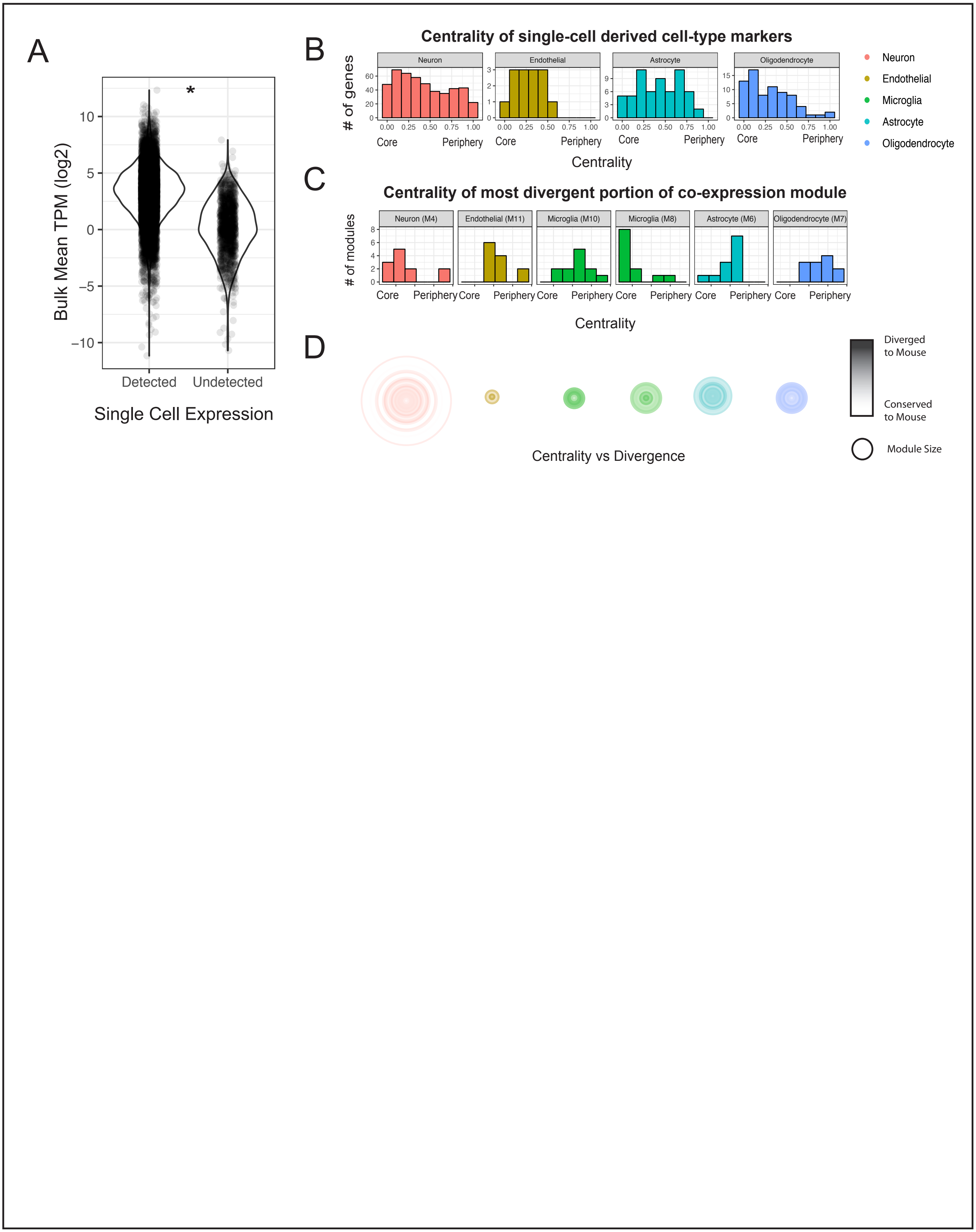

A
Bulk Mean TPM (log2)
10
5
0
−5
−10
*
Detected
Undetected
Single Cell Expression
B
Centrality of single-cell derived cell-type markers
# of genes
Neuron
Endothelial
Astrocyte
Oligodendrocyte
0.00
0.25
0.50
0.75
1.00
Core
Periphery
Centrality
Neuron
Endothelial
Microglia
Astrocyte
Oligodendrocyte
C
Centrality of most divergent portion of co-expression module
# of modules
Neuron (M4)
Endothelial (M11)
Microglia (M10)
Microglia (M8)
Astrocyte (M6)
Oligodendrocyte (M7)
Core
Periphery
Centrality
D
Diverged to Mouse
Conserved to Mouse
Module Size
Centrality vs Divergence

Supplemental Figure Legends

Figure S1: Assessing potential confounders in generation of module divergence scores

A) The number of samples (left) and studies (right) contributing to each regional network. Sample or study number does not drive regional divergence.

B) Regional divergence highly correlates with regional divergence having mitigated the effect of cell type module proportion in that region, indicating divergence is not driven by differing proportion of cell type modules.

C) Proportion of cell type modules in human and mouse stratified by brain region

D) Module preservation but not module divergence is affected by module size. Cell type specification is the strongest driver of module divergence.

E) Regional preservation scores are highly correlated with study-regressed regional preservation scores faceted by species.

Figure S2: Supervised preservation validates divergence of glial cell types in human

A-B) Module divergence scores of gene co-expression modules (far left) and cell-type marker gene lists (center/right) derived from human (A) and mouse (B). Cell type markers gene lists were derived from independent co-expression (middle left), single-cell (middle right) and sorting based experiments (far right). Mouse cell types did not show a strong divergence, apart from some neuronal modules derived from (Miller et al., 2008) which appeared to be of hypocretinergic fate. In human, glial cell types displayed strongest divergence.

C) GO enrichment for Whole-Brain consensus modules WB-M2, WB.M3 and WB.M9 which do not possess cell type annotation.

D) Module divergence scores of human cell-type markers gene lists. Divergence scores of marker lists are largely similar with the exception of sorting derived astrocytes and oligodendrocytes (zhang; triangle down) which do not appear to show strong divergence in co-expression between human and mouse. Cell type divergence scores from these three independent methods appear to mirror those from our generated co-expression modules (bottom).

E) Module preservation scores for consensus ‘Whole Brain’ modules measured in human and mouse studies subdivided into young adult (human 13-40 years; mouse 2-14 months) and aging adult (human >40 years; mouse >14 months) samples.

F) Young and aging adult module preservation scores for each study are compared to create a ratio score. Studies with a log2 ratio score >0 suggest that study is more preserved in aging brain, whereas studies with a ratio score <0 suggest that module is more preserved in young adult brain. Microglial consensus modules WB.M10 and WB.M8 show strong (+/- 0.75 ratio score) and significant (pval < 0.01) differences in preservation between aging states in mouse (denoted by *).

G) Cell type enrichment for microglial module sets WB.M8 and WB.M10. M10 represents a more canonical microglial signature whereas M8 represents a more activated glial state

H) Divergence index of cell types across brain regions. Only regional cell-type classes with significantly greater divergence in human or mouse are displayed. All cell types in cortex display significant (*; pval < 0.05) relative divergence, whereas other regions display limited asymmetric divergence.

Figure S3: Sequence divergence is correlated with transcriptional divergence at multiple levels

A) Gene annotations tested for divergence at the sequence level.

B) Module divergence plotted against module level sequence divergence for each gene annotation. Transcriptomic divergence generally correlates with sequence divergence across cell types but not for modules without cell type enrichment.

C) Module divergence plotted against the module OR score for enrichment of mutation intolerant genes. Transcriptomic divergence correlates with module enrichment of LoF intolerant genes (pLI ≥ 0.9) across cell types but not for modules without cell type enrichment. Evolutionarily preserved modules at the transcriptional level are enriched in LoF intolerant genes.

D) Module divergence plotted against averaged dN/dS score for each module. Transcriptomic divergence correlates with the dN/dS divergence metric across cell types but not for modules without cell type enrichment.

Figure S4: Species differences in co-expression is associated with species differences in expression levels

A) Module divergence plotted against the proportion of diverged genes within that module. Highly divergent modules possess a greater proportion of divergent genes (kMEdiv>0.2).

B) kME divergence of i) all assayed genes and ii) human cell type marker genes previously identified to be strongly diverged to mouse (Miller et al., 2010; Kelley et al., 2018).

C) kME divergence of the genes comprising the top 100 diverged gene pairs in each Whole Brain consensus module. Top genes show stronger divergence than background, as expected, with top glial genes showing stronger kME divergence than those from neuronal or non cell type modules.

D) Correlation of HSPA2 gene expression to oligodendrocyte marker gene expression in all human and mouse studies. HSPA2 shows strong (7x greater) and significantly greater correlation to these genes in human versus mouse.

E) kME distribution of human-upregulated (> 2 FC), human-downregulated (< 0.5 FC) and similarly expressed (0.67 < FC < 1.5) genes with species expression differences derived from bulk (solid line) and singe-cell (dashed) expression datasets. Distributions are shown for i) all genes (top) and ii) neuronal (WB-M4), endothelial (WB-M11), astrocytic (WB-M6) and oligodendrocytic (WB-M7) genes (bottom).

F) Human-Mouse expression difference in bulk- and single-cell data for cell-types captured in both human and mouse single cell datasets. Positive and significant (pval < 0.01) correlation between bulk and single-cell data with the line of best fit passing close to the origin supports the interpretation that intracellular changes in gene regulation, rather than cellular composition may drive species differences in expression.

G) Average expression of four genes for each cell-type cluster in human and mouse single cell data (Hodges et al., 2019). Each gene displays increased expression in human according to both bulk and single-cell expression data for the cell-type it is associated with.

H) Mean species FC expression differences for neuronal, oligodendrocyte, endothelial and astrocytic genes for single cell and bulk expression data. Human-Mouse expression differences are similar in both platforms. Neuronal genes tend to be downregulated in human according to both single cell and bulk expression.

Figure S5: The periphery of canonical cell type modules show greatest divergence of co-expression

A) Genes with low expression according to bulk sequencing are less likely to be detected in single-cell data.

B) Module centrality of genes defined as markers from single cell datasets (Lake et al., 2018). Single cell markers sit closer to the core of cell type co-expression modules.

C) The periphery of cell type modules show greater divergence than the module core. Genes of each WholeBrain cell type module were ordered by kME and partitioned into gene lists of ~ 50 genes. As with each GTEx module, co-expression preservation and divergence was assessed for each centrality gene list in each brain region and species. Histogram plots for each cell type module showing the centrality bin which showed greatest co-expression divergence for each brain region. Module divergence appears to be largest towards the periphery of co-expression networks for all canonical cell type modules. The activated microglial module appears to have greatest co-expression divergence in the network core.

D) Visual representation of module divergence relating to centrality. Darker shades represent stronger divergence. Each ring in a network represents an additional 50 genes from the core. The neuronal (M4) network is the largest, thus has the largest number of rings. All cell type modules have a relatively conserved core, with peripheral genes displaying divergence. The activated microglial module (WB-M8) displays increased divergence in the module core.
