# Supplementary material for: Evolutionary conservation and divergence of the human brain transcriptome
Source: Genome Biol. 2021 Jan 29;22:52. doi: 10.1186/s13059-020-02257-z (PMC7844938; doi:10.1186/s13059-020-02257-z)
Supplement: Supplementary file 13 — Additional file 13. Review history. [file 13059_2020_2257_MOESM13_ESM.docx]

Review History

**First round of review**

**Reviewer 1**

**Are you able to assess all statistics in the manuscript, including the appropriateness of statistical tests used?**

Yes, and I have assessed the statistics in my report.

**Comments to author:**

Pembroke et al. present an impressively comprehensive and illuminating study of the evolutionary conservation and divergence of region-specific human brain co-expression networks as compared to mouse and non-human primates. By evaluating the robustness of whole brain and regional co-expression modules constructed in human and mouse (based on GTEx data and a set of 30 studies, respectively) across a large number of independent datasets, the authors are able to quantify and decribe region-, cell-type-, species-, and gene-specific co-expression divergence patterns. The authors also investigate the role of DNA sequence divergence for divergent co-expression modules, and they confirm with single-cell RNA-seq data that species-specific differences in mean expression, rather than cell type proportion, seem to underlie gene divergences. Finally, the authors demonstrate the coherence of human co-expression modules with data from 3D human brain organoids.

The methodological approach by Pembroke et al. is as follows: (1) construct region-specific robust co-expression networks on human (12 regions) and mouse (7 regions) -> (2) use hierarchical merging across regions to create a consensus whole brain network for each species -> (3) identify co-expression modules and assess robustness within species with independent datasets, using the Zsum statistic with 25 permutations each -> (4) calculate upper quartile (UQ) across test datsets of all Zsum scores for human, mouse, and non-human primate independently, and calculate divergence scores (ratio of UQ Zsum scores) for each previously identified robust module -> (5) use divergence scores to investigate species-, region- and cell-type-specific divergences.

This is a thorough and dense manuscript, and there is a lot information presented in the series of complex, multi-panel figures. Taken together, their results highlight the care that must be taken when extrapolating brain co-expression information from mouse to humans, particularly for species-divergent genes that have been shown to have significant association with neurological disorders or psychiatric disease. I greatly appreciate their innovative use of publicly available data resources, as well as their multi-layered (bulk and single-cell transcriptome, sequence variation, in vivo and organoids, different aging states) investigation of co-expression divergence patterns. Overall, the manuscript is very well-written, and I only have some minor remarks, as noted below.

** Minor remarks and questions **

-- line 113: I believe there is a missing reference indicated in the parentheses => (pLI; ref)

-- line 762: the authors refer to Fig S1F, but there is no such figure

-- line 802: z.k and IAC are never defined. Do sample sizes in Table S1 reflect the full datasets, or the number of samples remaining after outlier removal? If not, it may be useful to include the information of number of samples used in the rWGCNA analysis after outlier removal in the Table S1.

-- lines 804-810: It was a unclear for me what linear regression the authors are referring to here (with respect to the technical/biological covariates) -- I assume they are either referring to the Combat batch correction referred to in the following section ("Network generation") or the study regression presented in Figure S1E, but this is a bit unclear.

-- I think it is important for the authors to appropriately cite the methods used in the paper, especially in the case of WGCNA (Langfelder and Horvath 2008) / robust WGNCA (Langfelder and Horvath, 2012) as it plays such a central role in the analysis. I also noted the use of Salmon (Patro et al., 2017) and biomaRt (Durinck et al., 2009), which were not cited. On line 799 the authors note using Salmon to quantify mouse RNA-seq data, but then mention using technical covariates from STAR and picard. For which datasets were these latter tools used?

-- line 817: just to clarify, by robust WGCNA do the authors mean WGCNA with biweight midcorrelation?

-- lines 822-834: the authors specify for the mouse network that the whole-brain consensus was created using the common set of genes and that the cut height was chosen to obtain ~20 modules. Was this not the procedure used for the human network as well?

-- lines 828-834: the authors describe the naming scheme for each module, but I still had trouble interpreting the names of some modules (e.g. in Figure 2A-left, how is WB.M6 interpreted? Whole brain, but what does "M6" refer to?)

-- lines 894-904: are there ever any cases of modules that have divergence scores < 0? (e.g., a human module that has greater preservation in mouse than in human test datasets?) Figure 2B seems to suggest that all divergence scores are nonnegative.

-- For all software used as well as R, version numbers should be cited to facilitate reproducibility. For WGCNA, were parameters set at default values (e.g., soft threshold power, minimum module size, etc)?

-- Comments on figures:

=> Some figure references in the text are incorrect; e.g. line 245 mentions Fig 3G and line 762 mentions Fig S1F but neither exist.

=> Figure 2A-left: do the black lines represent the UQ of the Zsum? It could also be helpful to the reader to recall in the caption that each point represents a test dataset. The caption says that error bars (in this figure and others) are 95% CI from "permuting across study", what does that mean? The Methods only describe module label permutations for calculating Zsum, line 640 of the Discussion mentions bootstrapping the effect of study, and line 765 of the Discussion mentions study-level permutations -- could the authors be a bit more precise about what was actually done?

=> Figures 2B and 3B: what does the star represent?

=> Figure 2D: regional divergence score averaged across all modules?

=> Figure 4B-right, the title should read (Primate specificity). Figure 4C, based on the caption the x-axis labels be H and P (for human and primate rather than M).

=> Figure 5C caption mentions a * denoting some divergent regions, but none appear in the plot.

** Discretionary remarks **

Overall, the figures are very nicely made and pack in a lot of information, but are also VERY dense. I wonder whether a handful of subfigures can be omitted or moved to supplementary materials to help streamline the graphical information.

**Reviewer 2**

**Are you able to assess all statistics in the manuscript, including the appropriateness of statistical tests used?**

Yes, and I have assessed the statistics in my report.

**Comments to author:**

This manuscript presented a cross-species comparative network analysis for the brain regions and found evolutionarily conserved and divergent co-expressed genes in the human brain. In particular, authors integrated large-scale public gene expression data, constructed and compared the gene co-expression networks of various brain regions across human, mouse, and non-human primate (NHP). Further, they clustered the co-expression networks into many gene co-expression modules for regions and species. Also, they did the enrichment analyses for modules, including GOs, cell-type marker genes, disease genes, etc., and annotate the modules with top enrichments. Using the modular preservation, they developed a score to quantify how each module is evolutionary divergent across the species, in terms of co-expression changes. They mainly focused on the cell type modules to link evolutionary divergence, disease, and functions to cell types (e.g., microglia). They also found that the divergence of the regulatory sequences of modular genes likely explains co-expression divergence. Finally, they validated some cell-type and disease human modules using human brain organoids for recapitulating modular co-expression patterns. In general, this study was novel and advanced the understanding of the gene co-expression evolution in the human brain at the cell-type level. The manuscript was written clearly and organized well. However, I have several comments as follows:

1. Orthologous genes across species. How did authors select orthologous genes? Human-mouse only or human-mouse-NHP? Also, did authors use one-to-one orthologs only or include one-to-many or many-to-many orthologs? The latter also contributes to the evolutionary divergence, and likely affects calculating preservation and divergence scores. Ideally, comparative gene network analyses should consider co-expression in each species and orthologous relationship across species together to get robust evolutionary gene modules; e.g., IsoRank, OrthoClust, etc.

2. Co-expression network construction. WGCNA transforms the gene correlation matrix to a topological overlap matrix (TOM) so that the transformed gene co-expression network is scale-free. This scale-free transformation can make the hub genes (many neighbors) stand out. However, it might be risky to wash out some co-expression connections between a small group of genes that potentially contribute to the brain region functions. Thus, the modular preservations between authors data and independent data might be driven by the "scale-free" topology rather than the biology of co-expression. I suggest that authors check if the hub genes represent some brain region-specific functions, rather than housekeeping functions. Also, the authors didn't provide important WGCNA parameters such as scale-free R2, min module size. How consistent were these parameters for getting modules using independent datasets?

3. Cell-type proportion. How did authors normalize the cell type proportions (Fig S1B)? Did they estimate proportions first by single cell deconvolution? Also, it is unclear how Fig S4E supports that the cell-type proportion is not driving the species gene expression differences. How did author calculate the kME values of genes using single cell data? Many figure captions do not match very well, such as "similar expression" on Fig S4E vs. "stable genes" in its caption.

4. Sequence divergence. The authors calculated the sequence divergence scores using 40 mammalian species, but the modular div. scores by three species. This was inconsistent. I am curious about how seq. div. scores will change if using human, mouse and NHP. Also, the div. scores of sequence region types vary at different scales. For instance, introns and promoters (2kb up TSS) are a narrow range from 0.98 - 1, which looks a constant number compared to other sequence regions change from 0.1 - 0.8. Do authors have any insights on high div. scores of introns?

5. Gene regulation. The authors focused on the enrichment and association analyses for the co-expression modules, barely providing any mechanistic insights. Since co-expression implies co-regulation, can authors identify any underlying gene regulatory mechanisms of co-expression divergence, especially for disease modules? This may tell novel evolutionary features of gene regulatory networks in the brain. For example, are there any master regulators (e.g., TFs, enhancers) co-regulate the co-expressed genes of the divergent modules or cross-species conserved modules?

6. Gene expression data processing. The authors transformed the gene expression data by log2(TPM+0.001). This step introduced the negative values from lowly expressed genes that may significantly affect gene co-expression network construction. Also, 0.001 seems an arbitrary number. Typically, log2(TPM+1) is widely used for keeping low expression levels unchanged. Furthermore, when calculating the fold changes of gene expression between human and mouse, the authors used TPM+0.01 which didn't have log2 transformation and added 0.01. The authors need to justify these data processing steps.

7. Many methods are not sufficiently described such as missing parameters for network construction and clustering, enrichment analysis, single cell data, etc.

**Reviewer 3**

**Are you able to assess all statistics in the manuscript, including the appropriateness of statistical tests used?**

Yes, and I have assessed the statistics in my report.

**Comments to author:**

The structure of the paper is well-developed and the analysis is comprehensive and provides many interesting findings. First, the analysis encompasses a very rich collection of datasets from multiple sources, brain regions, species and sequencing modalities. The authors further use module analysis to integrate these datasets and explore the divergences of cell type specific modules across species and their correlation with sequence divergences as well as the transcriptome of in vitro datasets. Last but not least, the authors build the connection between disease risk genes and divergent co-expression genes between human and mouse, providing foundational resources for disease modeling. However, the paper can still be improved in some aspects. Otherwise, I feel this paper makes an important contribution to the field and would be well placed in your journal.

1. The authors used "neuron" as a single group when doing the analysis. However, there are both excitatory and inhibitory neurons, probably with different levels of species divergences. When combining them together, the species differences could be neutralized. The author should indicate the rationale of merging these two cell types and the merging won't change the results.

2. In Fig 3F, it seems that correlation between neuronal modules and sequence divergences is also very low, similar to that between non cell type modules and sequence divergences. The author might indicate whether there are any differences among cell types in terms of the association of transcriptomic divergence and sequence divergences.

3. The author used kME divergences density distribution (figure S4E) to illustrate that cell-type proportion is not a major driver of gene expression differences. However, to provide more solid evidence, the authors might also indicate how kME of the same gene between bulk and single cell data are correlated.

4. Mislabeling of figures. For example, there is no figure 3G provided.

5. The authors collected over seven thousand samples from the public database. The biggest challenge is how to properly integrate them. As stated in their method, it is not quite convince, for example, the authors matched the human and mouse data set by simply matching their Ensembl gene ids. The more efficient approach is required to exclude more diverged genes between species. Besides, the recount2 software is recommended to process the enormous datasets in the same pipeline as possible.

6. Many uncertain stuffs in Figure 4. For examples, Fig 4B, typo, two "Human Specificity"; Fig 4B, no "Microglia (Homeostatic)" and "Microglia (Activated)" in the plot (The legend should correspond to the plot); Fig 4C, typo, "M" should be "P"; Fig 4E, "Microglia" is more primate-specific than its two subgroups "Microglia (Hom)" and "Microglia (Act)"?

**Authors Response**

**Point-by-point responses to the reviewers’ comments:**

Reviewers responses

Reviewer #1: Pembroke et al. present an impressively comprehensive and illuminating study of the evolutionary conservation and divergence of region-specific human brain co-expression networks as compared to mouse and non-human primates. By evaluating the robustness of whole brain and regional co-expression modules constructed in human and mouse (based on GTEx data and a set of 30 studies, respectively) across a large number of independent datasets, the authors are able to quantify and decribe (sic) region-, cell-type-, species-, and gene-specific co-expression divergence patterns. The authors also investigate the role of DNA sequence divergence for divergent co-expression modules, and they confirm with single-cell RNA-seq data that species-specific differences in mean expression, rather than cell type proportion, seem to underlie gene divergences. Finally, the authors demonstrate the coherence of human co-expression modules with data from 3D human brain organoids.

The methodological approach by Pembroke et al. is as follows: (1) construct region-specific robust co-expression networks on human (12 regions) and mouse (7 regions) -> (2) use hierarchical merging across regions to create a consensus whole brain network for each species -> (3) identify co-expression modules and assess robustness within species with independent datasets, using the Zsum statistic with 25 permutations each -> (4) calculate upper quartile (UQ) across test datsets (sic) of all Zsum scores for human, mouse, and non-human primate independently, and calculate divergence scores (ratio of UQ Zsum scores) for each previously identified robust module -> (5) use divergence scores to investigate species-, region- and cell-type-specific divergences.

This is a thorough and dense manuscript, and there is a lot information presented in the series of complex, multi-panel figures. Taken together, their results highlight the care that must be taken when extrapolating brain co-expression information from mouse to humans, particularly for species-divergent genes that have been shown to have significant association with neurological disorders or psychiatric disease. I greatly appreciate their innovative use of publicly available data resources, as well as their multi-layered (bulk and single-cell transcriptome, sequence variation, in vivo and organoids, different aging states) investigation of co-expression divergence patterns. Overall, the manuscript is very well-written, and I only have some minor remarks, as noted below.

*We thank the reviewer for their strongly positive sentiments -- “impressively comprehensive and illuminating” -- and their suggestions for minor improvements, which we address below.*

-- line 113: I believe there is a missing reference indicated in the parentheses => (pLI; ref)

*Thank you for pointing this out. We have inserted the reference.*

-- line 762: the authors refer to Fig S1F, but there is no such figure

*Thank you for identifying this mistake. The text should have referred to Fig S1E instead, so we have changed the label accordingly.*

-- line 802: z.k and IAC are never defined.

*Thanks. We have edited this line in the Methods section of the manuscript to include definitions.*

Do sample sizes in Table S1 reflect the full datasets, or the number of samples remaining after outlier removal? If not, it may be useful to include the information of number of samples used in the rWGCNA analysis after outlier removal in the Table S1.

*The sample sizes in Table S1 are the number of samples remaining after outlier removal and therefore reflect the number of samples used in WGCNA analysis.*

-- lines 804-810: It was a unclear for me what linear regression the authors are referring to here (with respect to the technical/biological covariates) -- I assume they are either referring to the Combat batch correction referred to in the following section ("Network generation") or the study regression presented in Figure S1E, but this is a bit unclear.

*Sorry for being unclear. The linear regression mentioned is independent of the ComBat batch correction or Study regression. Instead this “linear regression” is an independent step after outlier removal to remove unwanted technical and biological covariates from the gene expression data. We have rephrased the relevant text in the Methods to make this clearer.*

-- I think it is important for the authors to appropriately cite the methods used in the paper, especially in the case of WGCNA (Langfelder and Horvath 2008) / robust WGNCA (Langfelder and Horvath, 2012) as it plays such a central role in the analysis. I also noted the use of Salmon (Patro et al., 2017) and biomaRt (Durinck et al., 2009), which were not cited.

*We apologize for the oversight. We have added missing references for methodological steps including those mentioned.*

On line 799 the authors note using Salmon to quantify mouse RNA-seq data, but then mention using technical covariates from STAR and picard. For which datasets were these latter tools used?

*STAR and Picard metrics were utilized for all the mouse RNA-seq datasets. We have edited the relevant line in the Methods to make this clearer in the manuscript.*

-- line 817: just to clarify, by robust WGCNA do the authors mean WGCNA with biweight midcorrelation?

*To clarify, in our manuscript, robust WGCNA refers to creating a consensus network by using the consensus of 50 bootstrapped networks. We appreciate that traditionally, robust has been used to describe the use of biweight midcorrelation, for our analysis we used the default Pearson’s correlation. We have now specified what we mean by “robust” in the Methods section: “For each regional expression matrix, 50 signed co-expression networks (Topological Overlap Matrices (TOMs)) were generated using Pearson’s correlation from 50 independent bootstraps of the samples with each bootstrapped co-expression network using the same estimated power parameter.”*

-- lines 822-834: the authors specify for the mouse network that the whole-brain consensus was created using the common set of genes and that the cut height was chosen to obtain ~20 modules. Was this not the procedure used for the human network as well?

*The modules for the human network were generated as described in Hartl et al., BioRxiv 2019 (https://doi.org/10.1101/2020.03.05.965749) and were used as a foundation for this work. In that work, it was not necessary to subset the whole brain consensus network to the common set of genes, since all the underlying regional networks were already subset to the common set of genes.*

*In contrast, for this paper, the mouse regional networks were not subset for brain wide commonly expressed genes. This means that for mouse, if a gene is expressed in a brain region, it will be a part of that regional network, even if it is not expressed in another brain region. To create a consensus whole-brain network, regional networks must be of the same dimensions and possess the same number of genes) before merging. Therefore, for mouse networks, an additional step sub-setting the regional networks to the common genes across all regions was necessary.*

*A cut height resulting in 20 modules was chosen for the mouse networks as this is approximately the number of modules which was created for the human networks. This has been added to the Methods.*

-- lines 828-834: the authors describe the naming scheme for each module, but I still had trouble interpreting the names of some modules (e.g. in Figure 2A-left, how is WB.M6 interpreted? Whole brain, but what does "M6" refer to?)

*M refers to ‘Module’, and the number after is an arbitrary number which shouldn’t be interpreted based upon its value. To answer your specific example directly however, if a regional module is labelled “WB.M6” it is because it had a significant overlap with the consensus module “WB.M6” (which is enriched in astrocyte markers - therefore this regional module is also likely to be astrocytic in nature). We now clarify this further in the Methods.*

-- lines 894-904: are there ever any cases of modules that have divergence scores < 0? (e.g., a human module that has greater preservation in mouse than in human test datasets?) Figure 2B seems to suggest that all divergence scores are nonnegative.

*There were a small portion (13%) of modules that displayed negative divergence scores but were relatively small in magnitude and were therefore rounded up to 0 (no divergence) for conceptual simplicity and to allow downstream calculations. We have altered the Methods and Figure Legend sections to clarify this. These negatively divergent modules are likely a result of the normal distribution in divergence observed around 0 (no divergence), since their 95% confidence intervals overlap 0, and they were much smaller than those with positive scores.*

*Below we plot the divergence scores on a log2 scale (rather than subtracting 1) to emphasize values more preserved in the opposing species. Compared to the positive log2 divergence scores (mean=1.45, max=5.48) the magnitude of these n¬egative divergence scores was small (mean=-0.52, min=-1.4). 98% of human modules displayed positive divergence scores, with the few negative divergence scores residing in the relatively more preserved ‘neuronal’ and ‘non-cell type’ categories. For mouse modules, we observe a number of neuronal and non-cell type modules that display negative divergence.*

*Since mouse neuronal and non-cell type modules are highly preserved in human, we do not expect to observe strong divergence scores for these modules. Therefore, negatively divergent modules are likely a result of the normal distribution in divergence observed around 0 (no divergence). This is supported by the observation that all negatively diverged modules reside within two standard deviations of the mean for that cell type category. We now explain this more fully in the Methods.*

-- For all software used as well as R, version numbers should be cited to facilitate reproducibility. For WGCNA, were parameters set at default values (e.g., soft threshold power, minimum module size, etc)?

*We thank the reviewer for this suggestion. Version numbers have been added in the methods for the analytical software used. The methodological parameters used for WGCNA have now been added to the manuscript.*

-- Comments on figures:

=> Some figure references in the text are incorrect; e.g. line 245 mentions Fig 3G and line 762 mentions Fig S1F but neither exist.

*We apologize for the mislabeling. Fig 3G referred to Fig 3F (but now Fig 3G) and Fig S1F referred to Fig S1E. These have been amended in the text.*

=> Figure 2A-left: do the black lines represent the UQ of the Zsum?

*Yes, the black lines represent the UQ. This clarification has been added to the figure legend.*

It could also be helpful to the reader to recall in the caption that each point represents a test dataset.

*Thanks for the suggestion, we have added a title to the subplot and edited the figure legend to make this more apparent.*

The caption says that error bars (in this figure and others) are 95% CI from "permuting across study", what does that mean? The Methods only describe module label permutations for calculating Zsum, line 640 of the Discussion mentions bootstrapping the effect of study, and line 765 of the Discussion mentions study-level permutations -- could the authors be a bit more precise about what was actually done?

*I have added a short paragraph in the “Calculating module divergence scores” Methods section which describes this permutation process.*

*“95% confidence intervals were calculated for each divergence score, by permuting across study. For each permutation, Zsum scores were sampled with replacement and an UQ score generated which was subsequently used to calculate a module divergence score. For each module, we generated and ordered 1000 permuted divergence scores. The 25th and 975th divergence score was selected as the 95% CI.”*

=> Figures 2B and 3B: what does the star represent?

*The star is an asterix and represents significance, this clarification has been added to the figure legends.*

=> Figure 2D: regional divergence score averaged across all modules?

*Correct, I have clarified this in the figure legend.*

=> Figure 4B-right, the title should read (Primate specificity). Figure 4C, based on the caption the x-axis labels be H and P (for human and primate rather than M).

*Thanks for the catch. The labels and titles have now been corrected.*

=> Figure 5C caption mentions a * denoting some divergent regions, but none appear in the plot.

*The asterixs’ were present at the end of the region name on the x-axis, but I have also added them to the top of the figure to make them more apparent. (Note: Now Figure 6B)*

** Discretionary remarks **

Overall, the figures are very nicely made and pack in a lot of information, but are also VERY dense. I wonder whether a handful of subfigures can be omitted or moved to supplementary materials to help streamline the graphical information.

*We thank the reviewer for this suggestion. We agree that Figure 5 especially was very dense – we have moved one of the subpanels to Fig S4 and split the remainder of the figure into two (Fig 5 & 6). We also used this opportunity to add Transcription Factor Binding Site enrichment analysis to Fig 5. To make the figures less dense, we have also spread out some of the panels within Figure 3 & 4.*

Reviewer #2: This manuscript presented a cross-species comparative network analysis for the brain regions and found evolutionarily conserved and divergent co-expressed genes in the human brain. In particular, authors integrated large-scale public gene expression data, constructed and compared the gene co-expression networks of various brain regions across human, mouse, and non-human primate (NHP). Further, they clustered the co-expression networks into many gene co-expression modules for regions and species. Also, they did the enrichment analyses for modules, including GOs, cell-type marker genes, disease genes, etc., and annotate the modules with top enrichments. Using the modular preservation, they developed a score to quantify how each module is evolutionary divergent across the species, in terms of co-expression changes. They mainly focused on the cell type modules to link evolutionary divergence, disease, and functions to cell types (e.g., microglia). They also found that the divergence of the regulatory sequences of modular genes likely explains co-expression divergence. Finally, they validated some cell-type and disease human modules using human brain organoids for recapitulating modular co-expression patterns. In general, this study was novel and advanced the understanding of the gene co-expression evolution in the human brain at the cell-type level. The manuscript was written clearly and organized well. However, I have several comments as follows:

1. Orthologous genes across species. How did authors select orthologous genes? Human-mouse only or human-mouse-NHP? Also, did authors use one-to-one orthologs only or include one-to-many or many-to-many orthologs? The latter also contributes to the evolutionary divergence, and likely affects calculating preservation and divergence scores. Ideally, comparative gene network analyses should consider co-expression in each species and orthologous relationship across species together to get robust evolutionary gene modules; e.g., IsoRank, OrthoClust, etc.

*This is an interesting point. When converting expression data to the corresponding species, orthologous relationships between those two species were considered and only one-to-one ortholog genes were used. This was based on facilitating an easier and more direct analysis (one to one) coupled with the notion that those with differences in orthologs are already candidates for involvement in evolutionary differences and we hoped to discover new evolutionary distinctions using this analysis. We do appreciate that genes with one-to-many or many-to-many orthologous relationships contribute to divergence, and now have added some text in the discussion to address this issue. However, as these represent 6.0% and 1.2% (Human) and 7.9% and 2.1% (Mouse) of orthologous relationships respectively, these relationships would not likely strongly alter the biological findings reported in this manuscript and they could potentially limit or complicate the interpretability as well. In some ways, we view a comprehensive and careful analysis of genes with divergent orthologs as distinct from the current analysis, warranting its own treatment in future work. We have added our rationale to the manuscript.*

2. Co-expression network construction. WGCNA transforms the gene correlation matrix to a topological overlap matrix (TOM) so that the transformed gene co-expression network is scale-free. This scale-free transformation can make the hub genes (many neighbors) stand out. However, it might be risky to wash out some co-expression connections between a small group of genes that potentially contribute to the brain region functions. Thus, the modular preservations between authors data and independent data might be driven by the "scale-free" topology rather than the biology of co-expression. I suggest that authors check if the hub genes represent some brain region-specific functions, rather than housekeeping functions.

*This is an interesting issue in reference to hubs driving the relationships observed. We do agree that a scale-free topology assumption creates networks with the presence of hub genes with high connectivity. Many theoreticians and subsequent experimental follow up studies have demonstrated that molecular networks from all forms of life obey scale topology to a first approximation (Barbasi et al; doi:10.1038/nrg1272; Horvath et al; doi: 10.2202/1544-6115.1128 and others; also see this reference for alternative viewpoints https://doi.org/10.1038/s41467-019-08746-5). As the reviewer points out, this structure has the advantage that one can identify hubs, which are the most central genes within a network or module - although within this structure there is room for a hierarchical, modular structure, which we capitalize on for many of our studies including this one. However, we emphasize that the preservation analysis used in this manuscript is not only dependent on hubs, but rather uses composite Zsum scores based on two major factors, density statistics (how well connected, include both gene-gene correlation and hub status measures such as kME) and connectivity statistics, a component of which is also based on the correlation network and thus, do not depend entirely on scale free topology (https://doi.org/10.1371/journal.pcbi.1001057).*

*To confirm that our preservation scores are not driven by scale free topology, we assess the correlation between module preservation (Zsum) scores and module cor.cor scores – a metric based upon the gene-level correlation in correlation (*not* adjacency) matrices. The strong correlation (cor=0.69, pval < 1e-15) between Zsum and cor.cor scores indicate that preservation is not driven solely by differences in connectivity, but also raw co-expression.*

*In the analysis presented in our manuscript, we also computed gene-level divergence (kMEdiv) scores. This was calculated, in part, using the moduleEigengenes function which transforms the correlation matrix into an adjacency matrix. It is therefore informative to determine the extent our kMEdiv scores are associated with raw correlation divergence scores, which have not been derived from data with scale free topology.*

*To generate these correlation divergence (CORdiv) scores, we generated a correlation (*not* adjacency) matrix for the reference and test datasets. Then for each gene, we 1) calculated the correlation of correlations (cor.cor) between the reference and each test dataset, 2) calculated the mean cor.cor for both Human and Mouse, and 3) subtracted the Mouse mean cor.cor from the Human mean cor.cor. This CORdiv score is driven by gene gene edges and not affected by kME or hub status at all. We then assessed whether our kMEdiv scores were associated with these CORdiv scores. We observe a strong positive correlation (cor=0.53; pval < 1e-15) between the kME div scores and our newly generate¬d CORdiv scores. This indicates that hub status is not solely driving our gene level divergence metric.*

*Lastly, when assessing gene-level co-expression divergence, we observe that it is often the module periphery (non-hub genes) th¬¬at display the greatest divergence in co-expression (Fig S5C). Therefore, we can confidently say that module divergen¬ce is not only being driven by divergence of hub genes that may have arisen through scale free topology.*

Also, the authors didn't provide important WGCNA parameters such as scale-free R2, min module size. How consistent were these parameters for getting modules using independent datasets?

*We thank the reviewer for this suggestion. We have added these details to the manuscript. The minimum module size (50) remained constant while the power parameter ranged between 6-20.*

3. Cell-type proportion. How did authors normalize the cell type proportions (Fig S1B)? Did they estimate proportions first by single cell deconvolution?

*For clarification, we did not normalize by cell type proportion, rather we normalized the regional divergence scores by the proportion of cell type modules in each region.*

Also, it is unclear how Fig S4E supports that the cell-type proportion is not driving the species gene expression differences.

*We apologize for the mislabeling. Figure S4F & G (now H), but not S4E, should support that statement, it has now been corrected. For example, in Fig S4G (now H), as the FC expression differences observed in the bulk expression data are similar to that seen in the single-cell expression data, it suggests that intracellular regulation drives these species differences in expression.*

How did author calculate the kME values of genes using single cell data?

*We apologize for the confusion. kME divergence was calculated using the bulk sequencing data. The figure legend (Fig S4E) is referring to what dataset (“single” or “bulk”) a gene’s expression is determined to be human- or mouse- upregulated. We have rephrased the figure legend for clarity.*

Many figure captions do not match very well, such as "similar expression" on Fig S4E vs. "stable genes" in its caption.

*We thank the reviewer for pointing this out. The figure caption has been altered to match that of the figure legend.*

4. Sequence divergence. The ¬¬ calculated the sequence divergence scores using 40 mammalian species, but the modular div. scores by three species. This was inconsistent. I am curious about how seq. div. scores will change if using human, mouse and NHP.

*We thank the reviewer for this suggestion. We are not aware of any Human to Mouse ‘PhastCons’ or other relevant base pair resolution sequence divergence scores for which we can utilize for this analysis, therefore the PhastCons score which incorporates sequence divergence across 30 mammalian species was the most relevant datatype and is indicative of the general sequence divergence for these human genes. Note: in the manuscript we described the PhastCons score incorporating sequences across 40 species, when in fact it was across 30 species - this number has now been changed.*

*We have tried to address this using the available data on Human-Mouse sequence divergence for each gene and averaged across genes for each module. The DN/DS scores shows a positive correlation (cor=0.35; pval <0.01) with Human-Mouse module divergence similar to that of the PhastCons scores. We have added this figure and relevant text to the manuscript. To create space for this plot, an ancillary figure (then Fig S3C) subplot (and the corresponding text in the manuscript) was removed. We recognize that the DN/DS ratio is a metric for coding regions and does not include non-coding gene regions such as the promoter or UTRs.*

*We also want to clarify that the Module Divergence scores in these plots represent Human to Mouse divergence and does not consider the transcriptomic divergence to NHP.*

Also, the div. scores of sequence regio¬n types vary at different scales. For instance, introns and promoters (2kb up TSS) are a narrow range from 0.98 - 1, which looks a constant number compared to other sequence regions change from 0.1 - 0.8. Do authors have any insights on high div. scores of introns?

*We find it interesting that the correlation between transcriptomic and sequence divergence exists for different gene regions which experience differing levels of selection pressure. We expect that the strong divergence of introns reflect the fact that intron sequences are spliced out and do not have a functional role in post-transcriptional regulation or translation. While promoters (250bp upstream) show more sequence preservation, the 2kb upstream shows strong divergence. This likely reflects the fact that the majority of this 5’ 2kb upstream is non-functional and not under selection pressure.*

5. Gene regulation. The authors focused on the enrichment and association analyses for the co-expression modules, barely providing any mechanistic insights. Since co-expression implies co-regulation, can authors identify any underlying gene regulatory mechanisms of co-expression divergence, especially for disease modules? This may tell novel evolutionary features of gene regulatory networks in the brain. For example, are there any master regulators (e.g., TFs, enhancers) co-regulate the co-expressed genes of the divergent modules or cross-species conserved modules?

*We thank the reviewer for this suggestion. In an attempt to assess the gene regulatory mechanisms of co-expression divergence, we performed transcription factor binding site (TFBS) analysis upon the significantly diverged genes for each WB consensus cell-type module and have identified TFs which may be driving these divergent co-expression patterns. We have added such analysis to the manuscript and include enriched TFBS in Supplementary Table 5. For example, the binding site of TF PU.1 is 5.3 times more likely be in the 1000bp upstream region of diverged genes from the WB.M10 module than a background set of brain expressed genes. This indicates that the change in expression or function of this TF may have induced a different co-expression pattern of the genes it regulates. To assess whether a change in co-expression or expression of the TF itself may be driving the downstream effects, we sought to assess whether these implicated TFs had a higher kME divergence or expression difference than background. We found that these TFs do not show significantly greater 'correlation divergence' (FC=1.37, p=0.33) or less 'expression differences' (FC=0.76, p=0.09). This suggests that in general, Human-Mouse cell type divergence is not driven by evolutionary changes of expression or co-expression of the TFs themselves, but reflect the sequence of the target genes, which is consistent with our analyses shown above (#4). We have added this analysis to the Results section of the manuscript.*

6. Gene expression data processing. The authors transformed the gene expression data by log2(TPM+0.001). This step introduced the negative values from lowly expressed genes that may significantly affect gene co-expression network construction. Also, 0.001 seems an arbitrary number. Typically, log2(TPM+1) is widely used for keeping low expression levels unchanged.

*The reviewer raises a valid point. We note, however, the introduction of negative values, per se, should not affect gene correlation or network construction. We agree that log transformation of the expression data will often decrease the influence of highly expressed (and therefore variable genes) and increase the influence of lowly expressed genes. The constant added to TPM before log transformation affects the influence of lowly expressed genes and is somewhat arbitrary. The larger the constant, the less weighting given to the lower values. We agree that using a larger integer, such as 1, would reduce the effect of lowly expressed genes.*

*To assess how the constant added (1 vs 0.001) affects correlation, we created a new expression matrix using the transformation log2(TPM+1) and assessed the correlation of each gene between the two datasets. We display these correlations for BRNCTX (Human) and CTX (Mouse) below.*

Human	Mouse

*As expected, genes with lower expression display a reduced correlation between both datasets (as the value of the constant 1 is more influential). The average gene correlation between log2(TPM+1) and log2(TPM+0.001) was 0.98 (Human) and 0.94 (Mouse), with only 2% (Human) and 14% (Mouse) of genes with a correlation < 0.8, indicating a minimal effect of this constant. Furthermore, of these “low correlation” genes, 90% (Human) and 99% (Mouse) were classified in the “grey” module likely due to their low expression and were therefore not part of the downstream module analysis.*

*While the influence of lower values upon co-expression network construction could be further discussed, we do not feel the constant added (0.001 vs 1) effects network construction in any significant way, given the similarity of correlation (0.94-0.98 mean correlation) between the two sets and the fact that a large proportion (90-99%) of these affected genes (<0.8 cor) reside in the ‘grey’ (non-assigned) module.*

Furthermore, when calculating the fold changes of gene expression between human and mouse, the authors used TPM+0.01 which didn't have log2 transformation and added 0.01. The authors need to justify these data processing steps.

*Log2 transformation is recommended for correlation analysis to reduce the effect of highly expressed and variable genes. It is appropriate to use raw values to calculate fold change (FC) For example, Human TPM =10, Mouse TPM =5, the FC difference in expression would be 2 - rather than log2(10) / log2(5) = 1.43.*

*As previously mentioned by the reviewer, adding a constant before a transformation or calculation reduces the weighting of small numbers. Before addressing this concern, we must state that the constant added was not 0.01 as reported in the manuscript, but 0.1, and has now been adjusted. We are very pleased the reviewer commented on this so as to allow us to find this mislabeling.*

*The addition of the constant 0.1 was chosen to prevent aggregated FC scores due to extremely low mean TPM values (<0.1 TPM) while still allowing the illumination of human and mouse expression differences for lowly expressed genes (~0.1-1 TPM). We felt this was a more conservative approach, preventing influence of very low values observed in single cell datasets. We have added an explanation to this segment of the manuscript.*

7. Many methods are not sufficiently described such as missing parameters for network construction and clustering, enrichment analysis, single cell data, etc.

*We apologize and have added further methodological details including to the stages mentioned above.*

Reviewer #3: The structure of the paper is well-developed and the analysis is comprehensive and provides many interesting findings. First, the analysis encompasses a very rich collection of datasets from multiple sources, brain regions, species and sequencing modalities. The authors further use module analysis to integrate these datasets and explore the divergences of cell type specific modules across species and their correlation with sequence divergences as well as the transcriptome of in vitro datasets. Last but not least, the authors build the connection between disease risk genes and divergent co-expression genes between human and mouse, providing aspects. Otherwise, I feel this paper makes an important contribution to the field and would be well placed in your journal.

*We thank the reviewer for their recognition of the well-developed structure of the manuscript and comprehensive analysis with many interesting findings. We also appreciate the constructive criticism whose goal is to improve the paper in several respects.*

1. The authors used "neuron" as a single group when doing the analysis. However, there are both excitatory and inhibitory neurons, probably with different levels of species divergences. When combining them together, the species differences could be neutralized. The author should indicate the rationale of merging these two cell types and the merging won't change the results.

*The reviewer raises a good point and is one that we had previously investigated, but not added to the original manuscript. We had tested to see if any of our neuronal modules were enriched in inhibitory or excitatory neuronal markers, and then compared the module divergence between these two groups but found no significant difference between the two groups. As co-expression modules do not necessarily represent these subtypes perfectly, we also attempted to directly test the difference in divergence between these cell types by performing a ‘supervised module preservation’ approach using cortical inhibitory and excitatory neuronal markers derived from single cell analysis (Lake et al., 2018) and assessed their co-expression preservation between human and mouse. Again, there was no difference in divergence between the two neuronal subgroups.*

Module preservation	Supervised preservation

*As module divergence for our neuronal modules did not separate according to their enrichment for inhibitory or excitatory neuronal markers, we chose to keep the two groups together for cohesion. However, given the interest in this matter, we have added the relevant data (Fig 3D & Fig S2A) and text to the manuscript. Of note, we think that more single cell expression and gene regulation data across different species may be necessary to fully address this issue in the future.*

2. In Fig 3F (now Fig 3G), it seems that correlation between neuronal modules and sequence divergences is also very low, similar to that between non cell type modules and sequence divergences. The author might indicate whether there are any differences among cell types in terms of the association of transcriptomic divergence and sequence divergences.

*In general, there was no clear association between sequence and transcriptomic divergence within a cell-type. But the reviewer does raise an interesting point in that neuronal modules do not show strong transcriptomic divergence even at higher levels of promoter sequence divergence. We speculate that there is a similar mechanism to that of ribosomal modules that, even at stronger sequence divergence, the transcriptome of neuronal modules still displays limited divergence. These mechanisms may exist due to the essentiality of both ribosomal and neuronal function.*

3. The author used kME divergences density distribution (figure S4E) to illustrate that cell-type proportion is not a major driver of gene expression differences. However, to provide more solid evidence, the authors might also indicate how kME of the same gene between bulk and single cell data are correlated.

*We agree and we apologize, as this text was mislabeled, the text was intending to refer to figure S4F/G (now H). This figure shows the correlation of species expression FC differences derived from both bulk and single cell data. We suggest intracellular regulation is driving interspecies expression differences of cell-type genes given the similarity of species FC expression derived from both single-cell and bulk data.*

*We also want to clarify that it was not the kME correlations, but the mean expression values (and therefore interspecies expression FC differences) that were derived from both bulk and single cell data. We have rephrased the figure legend to make this clearer.*

4. Mislabeling of figures. For example, there is no figure 3G provided.

*We apologize and have now relabeled Figure 3G to Figure 3F (but now Figure 3G) as well as the other figure mislabeling.*

5. The authors collected over seven thousand samples from the public database. The biggest challenge is how to properly integrate them. As stated in their method, it is not quite convince,(sic) for example, the authors matched the human and mouse data set by simply matching their Ensembl gene ids. The more efficient approach is required to exclude more diverged genes between species.

*This is an important point. We matched human to NHP and mouse expression by using the corresponding one-to-one orthologs for that species, which is a fairly conventional approach for interspecies comparisons (Monaco et al; doi: 10.1186/s12862-015-0534-7, Huang et al; https://doi.org/10.1186/gb-2004-5-7-r47). We appreciate that some of these orthologous genes may diverge over evolutionary time and through this analysis we hope to identify the transcriptional differences that this divergence may induce. Therefore, excluding divergent genes from our network analysis may reduce the ability to identify true co-expression differences between the species.*

*..Besides, the recount2 software is recommended to process the enormous datasets in the same pipeline as possible.*

*We agree that if integrating different datasets into the same matrix, it is important to reduce the ‘researcher degrees of freedom’ by using same quantification and processing pipeline. To clarify, only our mouse RNA-seq discovery dataset required integration from different datasets. This is why we downloaded the raw fastqs for the mouse RNAseq and ran the data through the same pipeline. The remainder of our datasets were not ‘integrated’ per se. Instead, gene co-expression from the discovery dataset was tested in each test study independently. This means there is not a confound between study which may otherwise be present when combining datasets.*

*While recount2 certainly looks to be a valuable tool, we feel that it would not be completely suitable for this analysis as recount2 only offers human RNA-seq data, where this study utilized human, primate and mouse RNA-seq and array data. We thank the reviewer for pointing out this software which we believe will have value for future large scale studies integrating human data.*

6. Many uncertain stuffs in Figure 4. For examples, Fig 4B, typo, two "Human Specificity"; Fig 4B, no "Microglia (Homeostatic)" and "Microglia (Activated)" in the plot (The legend should correspond to the plot); Fig 4C, typo, "M" should be "P"; Fig 4E, "Microglia" is more primate-specific than its two subgroups "Microglia (Hom)" and "Microglia (Act)"?

*We apologize and have corrected the mislabeling in 4B and 4C.*

*The cell type color scheme in the legend of Fig 4B applies to the entire figure, not only the adjacent panel. There are a couple of instances in the figures where the color legend includes additional information which is not relevant to the adjacent figure. We have made clarifying comments in the legend to state if there is not a complete correspondence in the legend and directly adjacent figure.*

*A module is defined as “microglial” if it is enriched for microglial cell markers with an Odds Ratio (OR) above 5 and if the module does not show stronger enrichment for any other cell type. “Homeostatic” and “activated” microglia are defined by their membership in consensus modules WB.M10 and WB.M8 respectively. There is a single microglial module which does not fit into either of these two WB modules. Additionally, there are 4 modules which are part of the activated ‘WB.M8’ module whose cell-type enrichment is strongest for endothelial cells (which generally appear to be more human-specific). For these reasons, it is not necessary for the microglial score to sit between its two ‘subgroups’.*

**Second round of review**

**Reviewer 1**

The authors have provided thorough responses to all reviewer comments and have further strengthened their manuscript.

**Reviewer 2**

Thank authors for revising the manuscript. Authors have addressed many of my comments such as scale-free, orthologs, TF regulation for co-expression. However, I still have some concerns:

(1) for human-mouse expression changes of bulk vs. cell types (Fig S4F), actually, they are not significantly positively correlated (as claimed in the manuscript); e.g., cor=0.08 for neuron. Doesn’t this imply that multiple cell types contribute together to the bulk fc whereas each cell type contributes moderately (low correlation)?

(2) Also in Fig S4F, why is each gene’s bulk FC changing? For example, max bulk FC is close to 8 for oligo but less than 6 for Astrocyte and Endo.

(3) Microglia is missing in Fig S4F, H

(4) Minor: the manuscript includes several types of divergence such as sequence, module, co-expression, transcriptomics (bulk, single cell). I suggest authors define them upfront in a summary table.

(5) Minor: authors need to check the consistency of the terminology such as “dN/dS” vs. “DN/DS”.

**Authors Response**

**Point-by-point responses to the reviewers’ comments:**

Reviewer 2

1. For human-mouse expression changes of bulk vs. cell types (Fig S4F), actually, they are not significantly positively correlated (as claimed in the manuscript); e.g., cor=0.08 for neuron. Doesn’t this imply that multiple cell types contribute together to the bulk fc whereas each cell type contributes moderately (low correlation)?

*We emphasize that all of these interspecies FC relationships are indeed statistically significant and show positively correlations; this is the case for all cell types, as these correlation scores were both statistically significant (p<0.01) and showed positive correlation.*

*The correlation for neuron, while positive and significant, is much smaller than the others. There are many possible reasons for why these correlation scores were not stronger. For example, drop-outs in single cell data may produce zero values for either species - producing FC scores which are inaccurate or inflated (if null in one species) or zero (if null in both species). Differences in the samples and protocols for the bulk and sc sequencing may also underlie the differences in interspecies FC scores, as these data are collected from different labs using different protocols. The maximum expected correlation between bulk and single cell expression, even when using the exact same samples in the same lab is no more than 0.6-0.7. Given this information, these significant correlations are notable.*

*One key issue between bulk and single cell data, as the reviewer correctly states, is that bulk FC differences may be driven by many cell types – not just one. As these genes displayed are likely cell type markers (as they are members of canonical cell type modules), it is also possible that these genes are not strongly expressed in other cell types and therefore this suggestion will not be a factor for the reduced correlation. To test this, we compared the expression level in the relevant cell type to the expression in all the cell types combined. If a neuronal gene is expressed exclusively in neurons, it will have a percentage score of 100%. If a neuronal gene is expressed equally in Neurons, Endothelia, Astrocytes, Microglia and Oligodendrocytes – it will have a percentage score of 20%. We show that the neuronal markers are very specific to neuronal cell types in the single cell data, therefore the lower correlation is probably not for this reason. It is worth noting that a portion of genes of the oligodendrocyte and astrocyte module do appear to be expressed in other cell types according to the single cell data, therefore any discrepancy between the bulk and single cell data for these genes could be due to expression changes in these other cell types.*

*A possible reason for the neuronal correlation to be¬¬ smaller than the other cell types is the fact that neurons are a highly heterogeneous cell type and perhaps certain cell subpopulations are changing in their abundance or regulation between species. To assess the expression level in “neurons” we took the mean expression level across all the neuronal cell type clusters. Of these clusters, around half were “excitatory” and half were “inhibitory”. However, we know that the ratio of excitatory to inhibitory neurons is approximately 8:2. Therefore, our interspecies calculations for the single cell data may have preferentially weighed the expression change of inhibitory neurons. In order to compensate for that, we calculated a weighted mean, weighing the mean expression of Excitatory neurons by 0.8 and the Inhibitory neurons by 0.2. This additional step should make the single cell and bulk neuronal data more aligned. The weighted mean improved the correlation from 0.08 to 0.1. We anticipate that by creating true pseudo bulk samples by utilizing the number of cells in each cluster for all cell types, we would be able to increase the correlation - however this is outside the scope of our manuscript. We have replaced the previous non-weighted correlation with these data and this figure which more closely represents pooled bulk.*

*While there is a degree of noise, given that there is a significant positive correlation with these two data sources, and that the data points run close to the origin of the graph (no global shift in FC changes; we have now added a line of best fit and added clarifying text into the manuscript to further explain), we believe that intracellular changes may drive the interspecies differences observed in bulk data, rather than changes in cell type abundance. If there was a consistent global shift in FC across up or down the y axis – it would suggest that there was a global change in cell abundance between the species.*

2) Also in Fig S4F, why is each gene’s bulk FC changing? For example, max bulk FC is close to 8 for oligo but less than 6 for Astrocyte and Endo.

*We are not entirely sure what the reviewer means by ‘each gene’s bulk FC changing’. Each gene’s Human-Mouse bulk expression FC is only displayed for a single cell type – the cell type it was assigned to according to its module membership, so are unsure how the FC value can change.*

*If the reviewer is asking why the max bulk FC is 8 for Oligo and under 6 for Astrocytes and Endothelial. We believe that the range is very similar between these three cell types with only two genes out of hundreds showing slightly stronger FC interspecies difference. We notice, however, that the neurons do appear to have a narrower FC range, we believe this may be because they are more heterogeneous with neuronal genes being expressed across different subtypes.*

3) Microglia is missing in Fig S4F, H

*In our manuscript, we preface our single cell expression analysis with “For cell types captured in both human and mouse single-cell datasets …”. As the mouse single cell dataset utilized did not have expression data for microglia, the human-mouse FC expression difference could not be calculated for this cell type. We chose to use these specific datasets for both species as they were generated by the same institute, processed in the same manner and have previously been used for interspecies comparisons and therefore a good resource to explore and verify our results.*

4) Minor: the manuscript includes several types of divergence such as sequence, module, co-expression, transcriptomics (bulk, single cell). I suggest authors define them upfront in a summary table.

*We have now attempted to clarify these types of divergence in Figure 1 and its corresponding legend, where we define these different classes of divergence.*

*In this manuscript, we use the terms co-expression divergence and transcriptomic divergence interchangeably, so that transcriptomic divergence signifies differences in co-expression. In most cases, these refer to differences at the module level (module divergence) and gene level (kME divergence), unless otherwise noted. We also use different measures of sequence divergence or constraint (pLI, phastCons, dN/dS), which are labeled at each use, and refer to changes occurring at the module level overall, rather than the gene level, unless specified. “Bulk” & “single cell” labels are unrelated to transcriptomic ‘divergence’ but are used to calculate interspecies expression differences.*

5) Minor: authors need to check the consistency of the terminology such as “dN/dS” vs. “DN/DS”.

*We have now used the terminology “dN/dS” consistently throughout the manuscript. We have checked the manuscript for consistency of other terminology as well.*
